# Identification of the main glutamine and glutamate transporters in *Staphylococcus aureus* and their impact on c‐di‐AMP production

**DOI:** 10.1111/mmi.14479

**Published:** 2020-02-11

**Authors:** Merve S. Zeden, Igor Kviatkovski, Christopher F. Schuster, Vinai C. Thomas, Paul D. Fey, Angelika Gründling

**Affiliations:** ^1^ Section of Molecular Microbiology Medical Research Council Centre for Molecular Bacteriology and Infection Imperial College London London UK; ^2^ Department of Pathology and Microbiology University of Nebraska Medical Center Omaha NE USA

**Keywords:** AlsT, amino acid transport, c‐di‐AMP, GltS, glutamate transporter, glutamine transporter, OpuD, osmolyte transport, *Staphylococcus aureus*

## Abstract

A *Staphylococcus aureus* strain deleted for the c‐di‐AMP cyclase gene *dacA* is unable to survive in rich medium unless it acquires compensatory mutations. Previously identified mutations were in *opuD*, encoding the main glycine‐betaine transporter, and *alsT*, encoding a predicted amino acid transporter. Here, we show that inactivation of OpuD restores the cell size of a *dacA* mutant to near wild‐type (WT) size, while inactivation of AlsT does not. AlsT was identified as an efficient glutamine transporter, indicating that preventing glutamine uptake in rich medium rescues the growth of the *S. aureus dacA* mutant. In addition, GltS was identified as a glutamate transporter. By performing growth curves with WT, *alsT* and *gltS* mutant strains in defined medium supplemented with ammonium, glutamine or glutamate, we revealed that ammonium and glutamine, but not glutamate promote the growth of *S. aureus*. This suggests that besides ammonium also glutamine can serve as a nitrogen source under these conditions. Ammonium and uptake of glutamine via AlsT and hence likely a higher intracellular glutamine concentration inhibited c‐di‐AMP production, while glutamate uptake had no effect. These findings provide, besides the previously reported link between potassium and osmolyte uptake, a connection between nitrogen metabolism and c‐di‐AMP signalling in *S. aureus*.

## Graphical abstract

A large number of amino acid transporters and oligopeptide permeases are encoded in bacterial genomes. However, their actual substrate specificity and functions are hard to predict bioinformatically. In this study, we report that GltS and AlsT are main glutamate and glutamine transporters in *Staphylococcus aureus*, respectively, and show that glutamine and ammonium uptake inhibit the production of the nucleotide signalling molecule c‐di‐AMP.

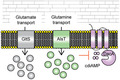

## 1 INTRODUCTION

In the human host, S*taphylococcus aureus* can grow in various tissues such as kidneys, bones, heart, soft tissues and lungs (Fridkin et al., 2005; Kluytmans, Belkum, & Verbrugh, 1997). Sensitive regulatory mechanisms enable this organism to rapidly respond to external stimuli and environmental changes. Amongst others, this allows bacteria to adapt their metabolism and utilize different carbon and nitrogen sources available in each specific niche (Crooke et al., 2013; Fridkin et al., 2005; Fuller et al., 2011; Halsey et al., 2017; Lehman et al., 2019; Richardson, Libby, & Fang, 2008; Spahich, Vitko, Thurlow, Temple, & Richardson, 2016; Vitko, Spahich, & Richardson, 2015).

Glucose is the preferred carbon source for *S. aureus,* but it can be limiting during infection due to the host immune response (Halsey et al., 2017; Kelly & O'Neill, 2015; Lehman et al., 2019; Spahich et al., 2016). In glucose‐limiting conditions, *S. aureus* instead catabolizes secondary carbon sources and amino acids, particularly glutamate and proline, which serve as major carbon sources during growth in the absence of glucose (Halsey et al., 2017). However, not much is known about amino acid uptake and catabolism in *S. aureus* and how the availability of certain nutrients can affect virulence factor expression and invasion of the host. While a large number of amino acid transporters and oligopeptide permeases can be identified bioinformatically, their actual substrate specificities and functions in *S. aureus* have not yet been studied in detail. Predicting the substrates for transporters bioinformatically remains challenging and hence such questions need to be addressed experimentally.

Secondary messenger molecules are crucial in allowing bacteria to rapidly adapt to different environmental and host cell niches (Hengge, 2009; Römling, 2008). There is now considerable evidence that one of these messengers, cyclic di‐adenosine monophosphate (c‐di‐AMP) plays a key role in osmoregulation in bacteria (Bai et al., 2014; Corrigan, Abbott, Burhenne, Kaever, & Gründling, 2011; Devaux et al., 2018; Fahmi, Faozia, Port, & Cho, 2019; Gundlach, Commichau, & Stülke, 2017; Gundlach, Herzberg, Hertel, et al., 2017; Pham et al., 2018; Pham & Turner, 2019; Quintana et al., 2019; Rocha, Teixeira‐Duarte, Jorge, & Morais‐Cabral, 2019; Teh, Dramsi, Tolker‐Nielsen, Yang, & Givskov, 2019; Whiteley et al., 2017; Whiteley, Pollock, & Portnoy, 2015; Witte et al., 2013; Zarrella, Metzger, & Bai, 2018; Zeden et al., 2018). c‐di‐AMP binds to and negatively regulates a number of different potassium and osmolyte importers (Chin et al., 2015; Corrigan et al., 2013; Devaux et al., 2018; Gundlach, Commichau, et al., 2017; Gundlach, Herzberg, Hertel, et al., 2017; Gundlach, Herzberg, Kaever, et al., 2017c; Huynh et al., 2016; Kim et al., 2015; Moscoso et al., 2015; Pham et al., 2018; Pham & Turner, 2019; Quintana et al., 2019; Rocha et al., 2019; Schuster et al., 2016; Zarrella et al., 2018). In many Firmicutes, c‐di‐AMP is essential for bacterial growth under standard rich medium growth conditions, but it is also toxic at high levels. Hence, the cellular levels must be tightly regulated (Corrigan et al., 2011; Corrigan, Bowman, Willis, Kaever, & Gründling, 2015; Gundlach et al., 2015; Mehne et al., 2013; Witte et al., 2013; Woodward, Iavarone, & Portnoy, 2010). In *S. aureus, Streptococcus agalactiae* and *Listeria monocytogenes,* deletion of *dacA* (also referred to as *cdaA* in many bacteria), coding for the diadenylate cyclase enzyme and responsible for the synthesis of c‐di‐AMP, was only possible in chemically defined medium (Devaux et al., 2018; Whiteley et al., 2015; Zeden et al., 2018), whereas in *Bacillus subtilis* all three c‐di‐AMP cyclases could only be inactivated in minimal medium also containing low amounts of potassium (Gundlach, Herzberg, Hertel, et al., 2017).

Previously, we found that inactivation of the main glycine betaine transporter OpuD (SAUSA300_1245) as well as the predicted amino acid transporter AlsT (SAUSA300_1252) allows an *S. aureus dacA* mutant to grow in rich medium in the absence of c‐di‐AMP (Zeden et al., 2018). In several other Firmicutes, including *B. subtilis, Lactococcus lactis, Streptococcus pneumoniae, S. agalactiae, Streptococcus pyogenes* and *L. monocytogenes*, inactivating mutations have also been identified in osmolyte and potassium transport systems that allow these bacteria to grow in the absence of c‐di‐AMP (Bai et al., 2014; Corrigan et al., 2011; Devaux et al., 2018; Fahmi et al., 2019; Gundlach, Commichau, et al., 2017; Gundlach, Herzberg, Hertel, et al., 2017; Pham et al., 2018; Pham & Turner, 2019; Quintana et al., 2019; Rocha et al., 2019; Teh et al., 2019; Whiteley et al., 2017, 2015; Witte et al., 2013; Zarrella et al., 2018; Zeden et al., 2018)*.* This suggests that potassium and osmolyte transporters are more active in the absence of c‐di‐AMP, resulting in the accumulation of toxic levels of potassium and osmolytes in the cell. Consistent with a key function of c‐di‐AMP in regulating the osmotic balance in the cell, we found that *S. aureus* cells show significant differences in cell size depending on their intracellular c‐di‐AMP levels (Corrigan et al., 2011; Zeden et al., 2018). Cells of strain LAC*gdpP, which have high c‐di‐AMP levels, show a decrease in cell size, while cells of strain LAC*dacA_G206S_, containing low levels of c‐di‐AMP, show an increase in cell size (Corrigan et al., 2011; Zeden et al., 2018). As c‐di‐AMP negatively regulates potassium and osmolyte uptake (Corrigan et al., 2013; Moscoso et al., 2015; Schuster et al., 2016), the increase in cell size is consistent with the hypothesis that an increase in potassium and osmolyte uptake and retention of water at reduced c‐di‐AMP levels leads to the observed increase in cell size. c‐di‐AMP levels affecting bacterial cell size has also been observed for other bacteria such as *S. pneumoniae* and *L. monocytogenes* (Bai et al., 2013; Commichau, Heidemann, Ficner, & Stülke, 2019).

AlsT is a predicted amino acid transporter and a correlation between cellular levels of c‐di‐AMP and the amino acids glutamate and glutamine has been reported for *B. subtilis* and *L. monocytogenes* (Gundlach, Commichau, et al., 2017; Gundlach et al., 2015; Sureka et al., 2014; Whiteley et al., 2017)*.* A twofold increase in cellular c‐di‐AMP levels was observed in *B. subtilis* when bacteria where grown in Spizizen minimal medium with glutamate (Glu) as compared to growth in the same medium with glutamine (Gln) as a nitrogen source (Gundlach et al., 2015). In *L. monocytogenes*, c‐di‐AMP was identified as a negative regulator of the key TCA cycle enzyme pyruvate carboxylase (Sureka et al., 2014)*.* Depletion of c‐di‐AMP resulted in an increased flux into the TCA cycle and as a consequence an increase in the cellular glutamine/glutamate pool (Sureka et al., 2014). In a *citZ* mutant, which lacks the TCA cycle enzyme citrate synthase and thus has an early block in the TCA cycle, the depletion of c‐di‐AMP no longer resulted in the accumulation of glutamate/glutamine in the cell (Sureka et al., 2014). Interestingly, an *L. monocytogenes dacA*/*citZ* double mutant was again viable in rich medium (Sureka et al., 2014; Whiteley et al., 2017).

As part of this study, we further investigated why inactivation of the main glycine‐betaine transporter OpuD and the predicted amino acid transporter AlsT allows *S. aureus* to grow in the absence of c‐di‐AMP in rich medium. We show that AlsT is a main glutamine transporter in *S. aureus* and that AlsT‐mediated glutamine uptake, and hence likely a high intracellular glutamine concentration, represses c‐di‐AMP production. Similarly, growth in ammonium‐containing defined medium but not in glutamate‐containing medium, repressed c‐di‐AMP production*.* The repression of c‐di‐AMP production was independent of the activity of the c‐di‐AMP phosphodiesterase GdpP and the predicted cyclase regulator YbbR. With this study, we not only provide a further link between the c‐di‐AMP signalling network and osmotic regulation in bacterial cells but also with the uptake of specific nitrogen sources and amino acids in *S. aureus.*

## 2 RESULTS

### 2.1 Inactivation of OpuD but not AlsT reduces the cell size of an *S. aureus dacA* mutant

**Figure 1:**
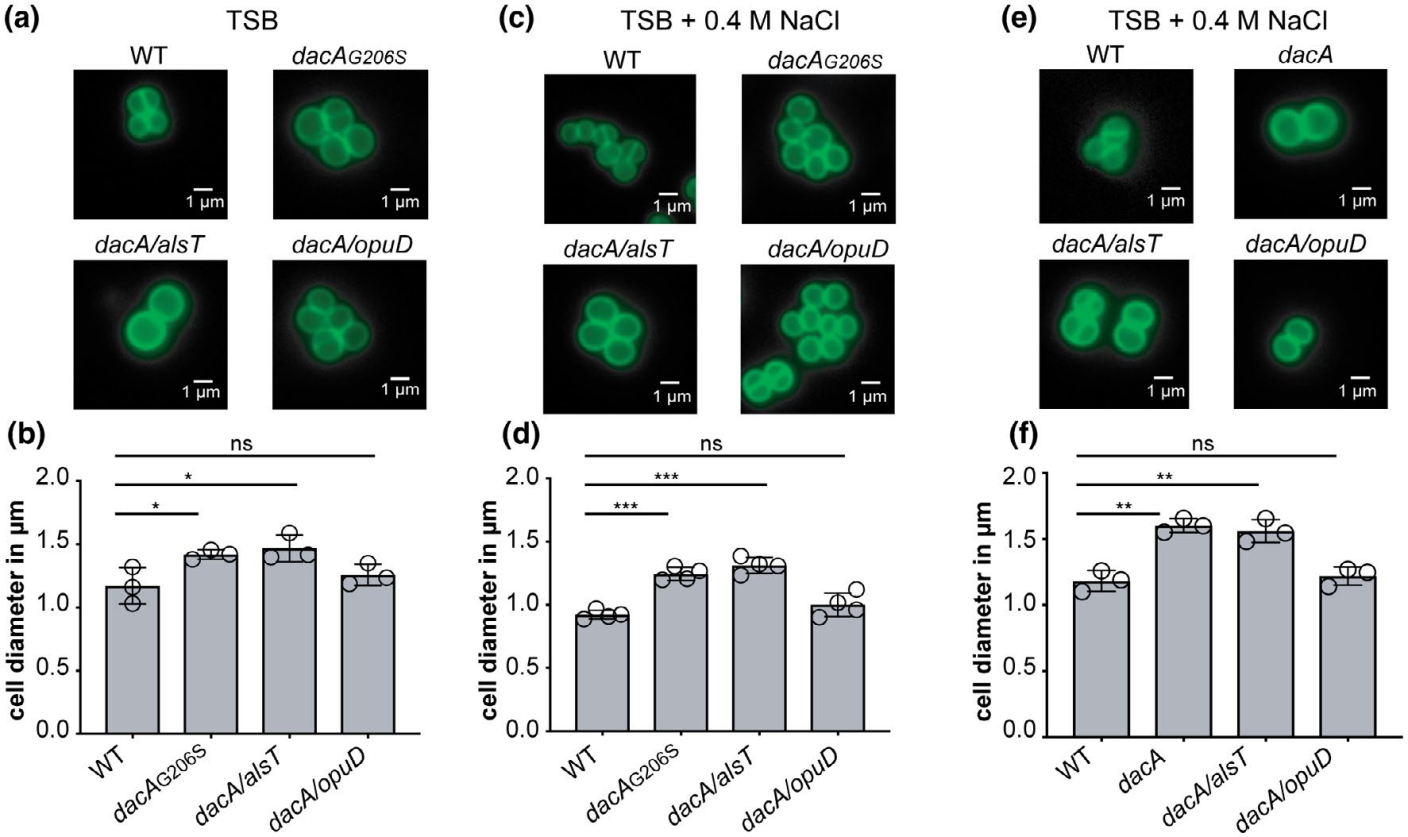
Inactivation of the glycine betaine transporter OpuD rescues the cell size of *S. aureus dacA* mutant bacteria. (a, c, e) Microscopy images of *S. aureus* cells stained with BODIPY‐labelled vancomycin. Cultures of *S. aureus* LAC* (WT), LAC*dacA_G206S_ (*dacA*_G206S_) (panels A and C only), LAC*dacA::kan (*dacA*) (panel E only) and the suppressor strains LAC*dacA/alsT (*dacA/alsT*) and LAC*dacA/opuD (*dacA/opuD*) were grown in (A) TSB or (C and E) TSB 0.4 M NaCl medium and subsequently stained with fluorescently labelled vancomycin. The bacteria were then viewed using a fluorescent microscope and representative images are shown. Scale bars are 1 µm. (b, d, f) Bacterial cell diameter measurements. The diameters of nondividing bacterial cells were measured as described in the Materials and Method section for *S. aureus* strains grown in (b) TSB or grown in (d and f) TSB 0.4 M NaCl medium. The diameters of 50 cells were determined and the average diameter calculated. The experiment was performed in triplicate (b and f) or quadruplicate (d) and the averages and SDs of the average cell diameters plotted. For statistical analysis, one‐way ANOVAs followed by Dunnett's multiple comparison tests were performed (ns = not significant, * = *p* < .01, ** = *p* < .001, *** = *p* < .0001)

In previous work, we reported a correlation between the cell size and c‐di‐AMP levels in *S. aureus*: bacteria with high c‐di‐AMP level are smaller, whereas bacteria with low c‐di‐AMP levels (strain LAC*dacA_G206S_) are larger as compared to wild‐type (WT) bacteria (Corrigan et al., 2011; Zeden et al., 2018). We also reported that inactivating mutations in *opuD* (*SAUSA300_1245*) coding for the main glycine betaine osmolyte transporter and *alsT* (*SAUSA300_1252*) coding for a predicted amino acid sodium symporter, rescue the growth defect observed for the c‐di‐AMP negative *S. aureus* strain LAC*dacA::kan in the rich medium TSB (Zeden et al., 2018). Here, we investigated further the mechanism by which the growth defect of the *dacA* mutant strain is rescued in the LAC*dacA/opuD and LAC*dacA/alsT suppressor strains. Initially, we compared the cell size of bacteria from the suppressor strains LAC*dacA/opuD and LAC*dacA/alsT to that of WT LAC* and the low c‐di‐AMP level strain LAC*dacA_G206S_ after growth in the rich medium TSB. As expected, the bacteria with low levels of c‐di‐AMP showed an increase in cell size as compared to WT bacteria (Figure 1a,b). While a similar increase in cell size was still observed for bacteria of strain LAC*dacA/alsT, the cell size of LAC*dacA/opuD bacteria, while still increased as compared to the WT, was significantly smaller as compared to the low‐level LAC*dacA_G206S_ strain (Figure 1a,b). Because regular TSB medium is not suitable for the growth of the c‐di‐AMP null strain LAC*dacA::kan, bacterial cell sizes were also determined following growth in TSB medium supplemented with 0.4 M NaCl, which is permissive for the growth of the *dacA* mutant (Figure 1c–f). Similar to what was observed for the low c‐di‐AMP level *dacA_G206S_* mutant strain, the size of bacteria from the c‐di‐AMP null strain LAC*dacA::kan was significantly increased compared to WT bacteria. As observed before, the cell size was not rescued for bacteria of the LAC*dacA/alsT suppressor strain (Figure 1c–f). On the other hand, the size of LAC*dacA/opuD bacteria was similar to that of WT bacteria (Figure 1c–f). Taken together, the observed differences in cell size indicate that the underlying molecular mechanisms enabling the *opuD* and *alsT* mutant strains to survive in the absence of c‐di‐AMP in rich medium might be different.

### 2.2 AlsT is a glutamine transporter in *S. aureus*

**Figure 2:**
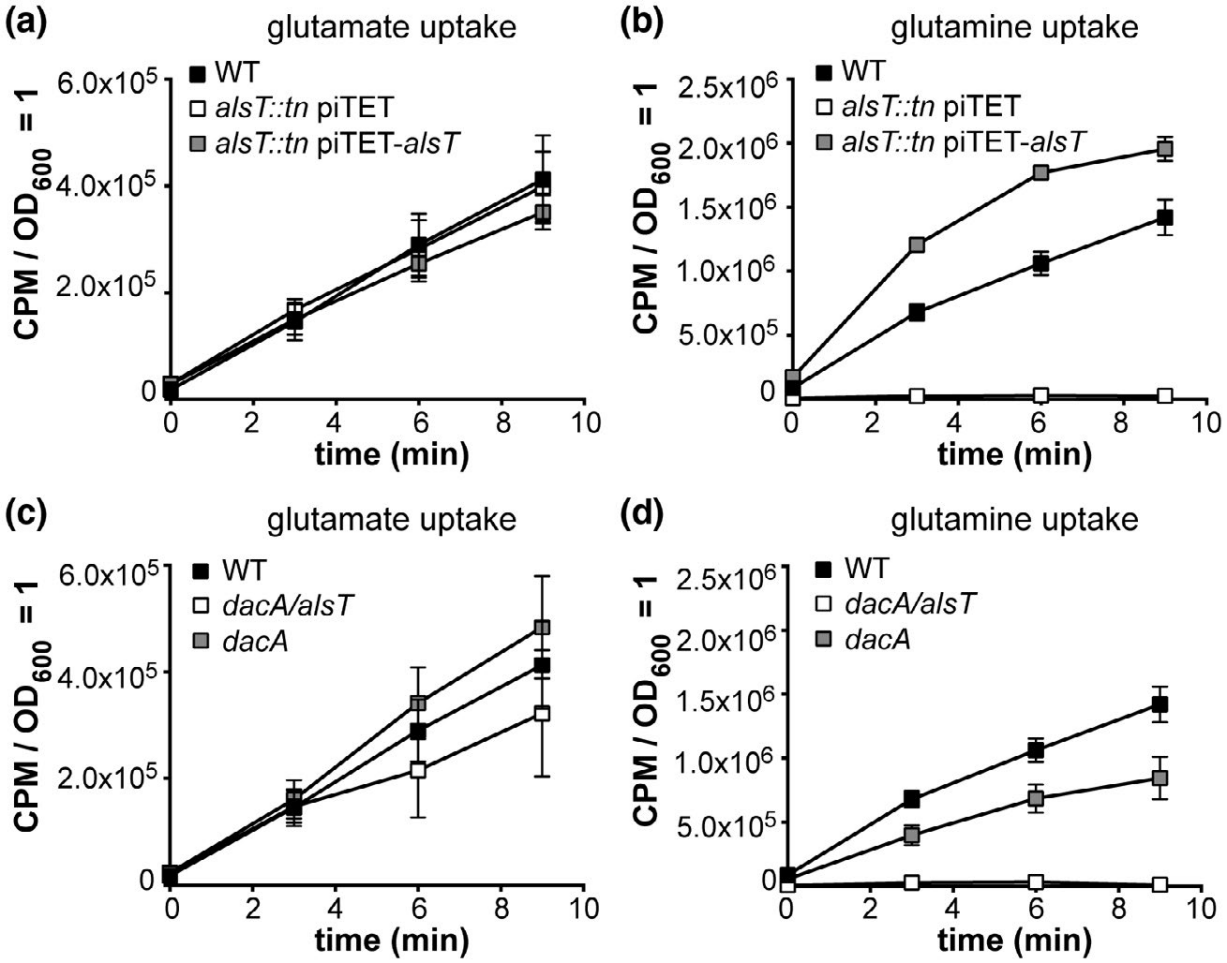
AlsT is a glutamine transporter in *S. aureus*. (a–d) Amino acid uptake assays. (a, b) *S. aureus* strain LAC* (WT), the *alsT* mutant LAC*alsT::tn piTET (*alsT::tn* piTET) and the complementation strain LAC*alsT::tn piTET‐*alsT* (*alsT::tn* piTET‐*alsT*) were grown to mid‐log phase in the glucose defined medium GDM + Glu+NH_3_, which was supplemented with 200 ng/ml Atet for the strains containing plasmids. Subsequently, radiolabelled (a) glutamate or (b) glutamine was added to culture aliquots, samples removed and filtered at the indicated time points and the radioactivity accumulated in the cells measured. The average values and SDs from three (b, d) or four (a, c) experiments were plotted. (c, d) the same uptake assay experiment was performed as described in (a, b) but using *S. aureus* strains LAC*dacA::kan (*dacA*) and LAC*dacA/alsT (*dacA/alsT*). The amino acid uptake curves for the LAC* (WT) strain are the same as shown in panels A and B, as all strains were grown and processed at the same time

AlsT (SAUSA300_1252) is a predicted amino acid transporter and annotated in the InterPro database (www.ebi.ac.uk/interpro) as alanine/sodium symporter. However, in a previous study no difference in the uptake of radiolabelled alanine was detected between a WT and the LAC*dacA/alsT mutant strain (Zeden et al., 2018), indicating that AlsT is not an alanine transporter. To identify potential substrates for the *S. aureus* AlsT transporter, we initially followed the depletion of different amino acids from the culture supernatant during the growth of the WT LAC* strain and the isogenic *alsT* transposon mutant strain LAC*alsT::tn in TSB medium, where both strains exhibit similar growth rates (Figure S1a). However, no drastic differences in the uptake of the different amino acids could be observed between the WT and *alsT* mutant strains (Figure S1). Of note, using this method, tryptophan uptake cannot be measured and it is also not possible to distinguish between glutamine/glutamate or asparagine/aspartate utilization. Next, a more detailed bioinformatics analysis was performed to identify potential AlsT substrates. A BlastP search against the *B. subtilis* 168 genome led to the identification of four proteins showing significant homology to the *S. aureus* AlsT (SAUSA300_1252) protein, namely AlsT (e‐value: e‐166), GlnT (e‐value: e‐149), YrbD (e‐value: e‐117) and YflA (e‐value: 2e‐72). Also in *S. aureus,* a second AlsT homologue, SAUSA300_0914 (e‐value 9e‐108), could be identified (Figure S2), which is encoded at a different chromosomal region. AlsT is annotated in *B. subtilis* as a potential glutamine sodium symporter (Zhu & Stülke, 2018), but to the best of our knowledge, this has not yet been experimentally verified. To test if *S. aureus* AlsT is a potential glutamine or glutamate transporter, uptake assays were performed with radiolabelled glutamine and glutamate using the WT *S. aureus* strain LAC*, the *alsT* mutant LAC*alsT::tn piTET and the complementation strain LAC*alsT::tn piTET‐*alsT*. Uptake of glutamine, but not of glutamate, was severely reduced in the *alsT* mutant when compared to the WT strain (Figure 2a,b). This defect was restored upon expression of *alsT* in the complementation strain (Figure 2b). To confirm that *alsT* functions as main glutamine transporter also in the LAC*dacA/alsT suppressor strain, uptake assays were also performed with strain LAC*dacA/alsT along with the WT LAC* and LAC*dacA::kan control strains (Figure 2c,d). Similar as observed for the *alsT* single mutant, glutamate uptake was only marginally affected in strain LAC*dacA/alsT (Figure 2c), whereas glutamine uptake was severely reduced in strain LAC*dacA/alsT when compared to the control strains (Figure 2d). These data suggest that under the uptake assay conditions tested, AlsT functions as the main glutamine transporter in *S. aureus.* Our data further suggest that *S. aureus* cells that are unable to produce c‐di‐AMP can survive in rich medium such as TSB, when glutamine uptake is reduced or blocked. However, we cannot formally exclude that AlsT is able to transport other amino acid or substrates present in rich medium.

### 2.3 Investigating the contribution of SAUSA300_0914 and GlnQ to glutamine and glutamate transport in *S. aureus*

**Figure 3:**
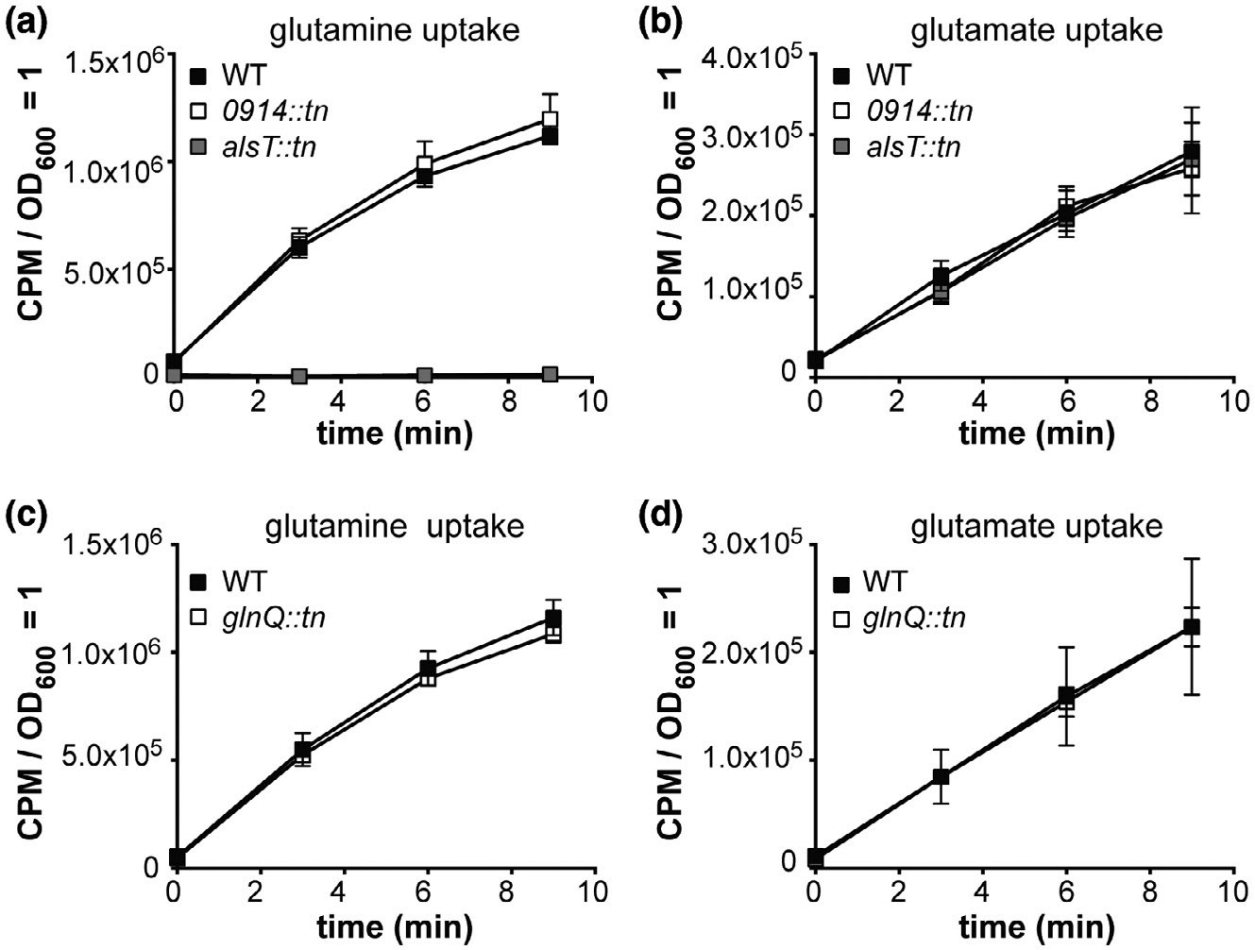
LAC*0914::tn and LAC*glnQ::tn strains do not show a defect in glutamine or glutamate uptake. Amino acid uptake assays. (a, b) *S. aureus* strains LAC* (WT), LAC*0914::tn and LAC*alsT::tn were grown to mid‐log phase in GDM + Glu+NH_3_. Subsequently, radiolabelled (a) glutamine or (b) glutamate was added to culture aliquots, samples removed and filtered at the indicated time points and the radioactivity accumulated in the cells measured. The average values and SDs from three experiments were plotted. (c, d) Amino acid uptake assays were performed and the data plotted as described in panels (a) and (b), but using *S. aureus* strains LAC* (WT) and LAC*glnQ::tn (*glnQ::tn*)

*S. aureus SAUSA300_0914* codes for a predicted amino acid symporter, which shows 41% identity to the *S. aureus* AlsT protein. After assigning AlsT a function as glutamine transporter, we wanted to test if SAUSA300_0914 might also play a role in glutamine or glutamate transport. To this end, strain LAC*0914::tn was constructed by transducing the genomic region from the NMTL strain NE1463 (Fey et al., 2013) containing a transposon insertion in *SAUSA300_0914* into the *S. aureus* LAC* background. Subsequently, the uptake of radiolabelled glutamine and glutamate was assessed (Figure 3a,b). No significant differences in the uptake of these amino acids was observed between WT LAC* and strain LAC*0914::tn, showing that SAUSA300_0914 does not function as a major glutamine or glutamate transporter under our assay conditions (Figure 3a,b). AlsT and SAUSA300_0914 are members of the amino acid/sodium symporter family of transporters, which are single, multimembrane spanning proteins. Besides this type of transporter, GlnPQ‐type ABC transporters play a major role in glutamine and glutamate transport in other bacteria (Schuurman‐Wolters & Poolman, 2005). *S. aureus* contains a *glnPQ (SAUSA300_1808*–*SAUSA300_1807)* operon with *glnP* coding for a substrate‐binding domain‐permease fusion protein and *glnQ* coding for the cytoplasmic nucleotide‐binding ATPase domain. The results from a previous study suggested that this transporter functions as glutamine transporter in *S. aureus*, as a *glnP* mutant was more resistant to the toxic glutamine analogue γ‐L‐glutamyl hydrazide (Zhu et al., 2009). To assess the contribution of the GlnPQ transporter to glutamine and glutamate transport in *S. aureus* LAC* under our assay and growth conditions*,* the strain LAC*glnQ::tn was generated by transducing the *glnQ::tn* region from the NMTL strain NE153 (Fey et al., 2013) into the LAC* background. The resulting *glnQ* mutant strain LAC*glnQ::tn displayed no difference in glutamine or glutamate uptake compared to WT LAC* (Figure 3c,d). This indicates that the ABC transporter GlnPQ does not function under our assay conditions and in the *S. aureus* LAC* strain background as a main glutamate or glutamine transporter. However, we cannot exclude that SAUSA300_0914 and GlnPQ could still function as glutamine or glutamate transporters under different growth conditions.

### 2.4 Inactivation of AlsT but not SAUSA300_0914 or GlnQ reduces the susceptibility of *S. aureus* to the toxic glutamine analogue γ‐L‐glutamyl hydrazide

**Figure 4:**
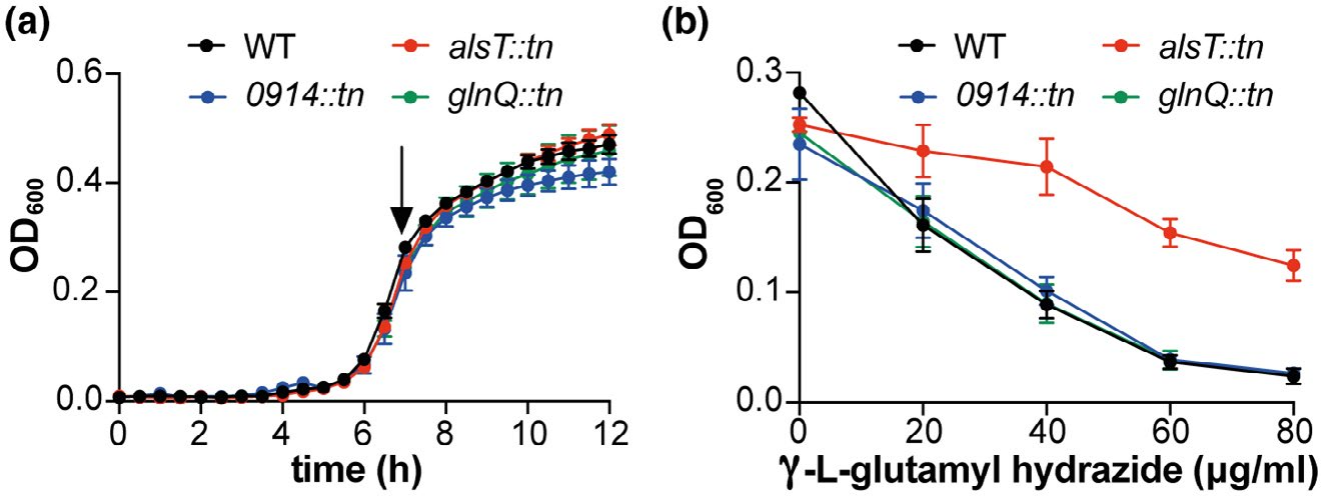
LAC*alsT::tn shows increased resistance to the toxic glutamine analogue γ‐L‐glutamyl hydrazide. (a) Bacterial growth curves. *S. aureus* strains LAC* (WT), LAC*alsT::tn, LAC*0914::tn and LAC*glnQ::tn were grown in GDM + NH_3_ medium in 96‐well plates and OD_600_ reading determined over 12 hr. The average OD_600_ values and SDs from three independent biological replicates were plotted. (b) γ‐L‐glutamyl hydrazide susceptibility assay. The same *S. aureus* strains as in (a) were grown in GDM + NH_3_ medium in the absence or presence of γ‐L‐glutamyl hydrazide at a final concentration of 20, 40, 60 or 80 μg/ml. OD_600_ reading were determined over 12 hr and the complete growth curves are shown in Figure S3. In this graph, the average OD_600_ values from the 7 hr time point (marked with an arrow in (a)) and SDs from three biological replicates were plotted against the γ‐L‐glutamyl hydrazide concentration in the growth medium

To further validate the findings from the uptake assays and verify that AlsT is the main glutamine transporter, we performed growth curves in the presence of increasing concentrations of the toxic glutamine analogue γ‐L‐glutamyl hydrazide with the WT and LAC*alsT::tn mutant strains. Strains LAC*0914::tn and LAC*glnQ::tn were also included in these assays, to uncover a potential low‐level glutamine uptake activity for the SAUSA300_0914 and GlnPQ transporters. Strains defective in taking up this glutamine analogue are expected to show reduced susceptibility to this toxic compound. In the absence of the compound, all strains grew similarly in the chemically defined medium used for this assay (Figure 4a). As expected, addition of γ‐L‐glutamyl hydrazide reduced the growth of the WT LAC* strain, in a dose‐deponent manner (Figures 4b and S3). Similar growth inhibition curves to that of the WT strain were obtained for strains LAC*0914::tn and LAC*glnQ::tn, while strain LAC*alsT::tn showed increased resistance to the compound (Figures 4b and S3). These findings support our earlier conclusion that AlsT is the main glutamine transporter in *S. aureus* LAC* under our assay conditions, while GlnPQ and SAUSA300_0914 are either unable to take up glutamine or play only a minor role in its uptake under our growth conditions.

### 2.5 GltS (SAUSA300_2291) is a glutamate transporter in *S. aureus*

**Figure 5:**
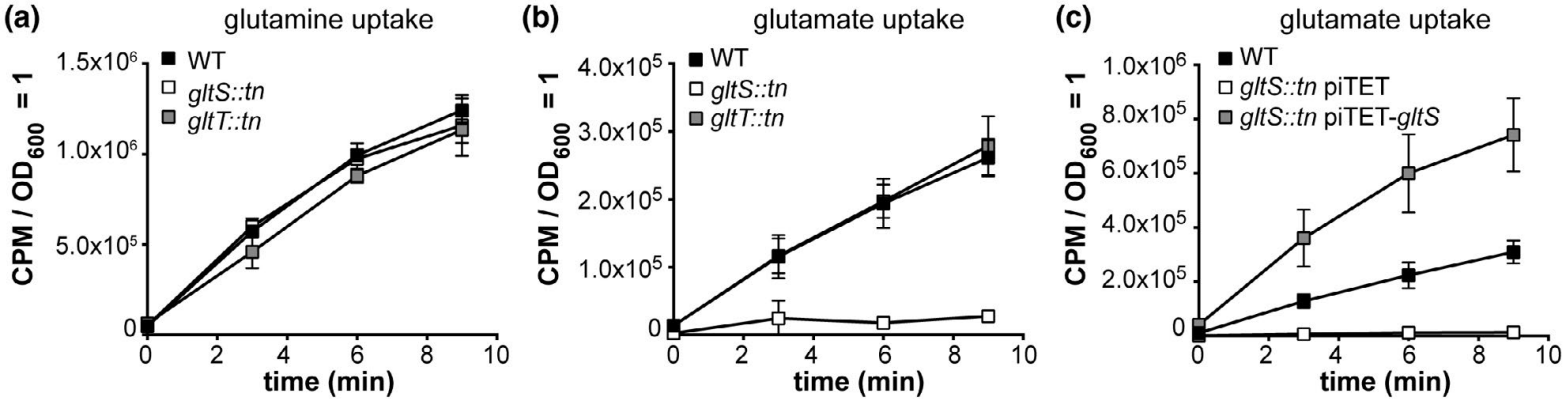
GltS is a glutamate transporter in *S. aureus*. Amino acid uptake assays. (a,b) *S. aureus* strains LAC* (WT), LAC*gltT::tn and LAC*gltS::tn were grown to mid‐log phase in GDM + Glu+NH_3_. Subsequently, radiolabelled (a) glutamine or (b) glutamate was added to culture aliquots, samples removed and filtered at the indicated time points and the radioactivity accumulated in the cells measured. (c) Same as (b) but using *S. aureus* strains LAC* (WT), LAC*gltS::tn piTET and the complementation strain LAC*gltS::tn piTET‐*gltS* and supplementing the GDM + Glu+NH_3_ medium with 200 ng/µl Atet. The average values and SDs from three experiments were plotted

*S. aureus* does not only take up glutamine but also shows robust glutamate uptake (Figures 2 and 3). However, none of the transporters (AlsT, SAUSA300_0914 and GlnPQ) investigated so far plays a major role in glutamate uptake under our growth conditions. In *B. subtilis* GltT, belonging to the dicarboxylate/amino acid cation symporter (DAACS) family of proteins, is a major high‐affinity Na^+^‐coupled glutamate/aspartate symporter and can also mediate the uptake of glyphosate (Wicke et al., 2019; Zaprasis, Bleisteiner, Kerres, Hoffmann, & Bremer, 2015). Two paralogs, DctP and GltP, are found in *B. subtilis* of which GltP has also been shown to be a glutamate transporter (Tolner, Ubbink‐Kok, Poolman, & Konings, 1995). The *S. aureus* protein SAUSA300_2329 (from here on referred to as GltT) shows a high degree of similarity (52% identity) to the *B. subtilis* GltT protein. In addition, SAUSA300_2291 (from here on referred to as GltS) is annotated in UniProt (www.uniprot.org) as a potential glutamate transporter in *S. aureus*. To experimentally test if GltT or GltS impact glutamate transport in *S. aureus*, strains LAC*gltT::tn and LAC*gltS::tn were constructed by moving the respective *gltT and gltS* transposon insertion regions from the NMTL strains NE566 and NE560 (Fey et al., 2013) into the LAC* strain background. Next, the uptake of radiolabelled glutamine and glutamate was assessed for WT LAC* and strains LAC* *gltT::tn* and LAC* *gltS::tn*. No difference in the uptake of glutamine was observed between the strains (Figure 5a) and in the case of LAC*gltT::tn, also no difference in the uptake of glutamate was observed (Figure 5b). However, a significant reduction in glutamate uptake was observed for strain LAC*gltS::tn when compared to the WT strain (Figure 5b). The glutamate uptake defect could be restored in a complementation strain harbouring plasmid piTET‐*gltS* allowing for inducible *gltS* expression (Figure 5c). Indeed, increased glutamate uptake was observed in the complementation strain, indicating increased *gltS* expression in the complementation strain as compared to the WT strain. Taken together, these data indicate that under the growth conditions tested, GltS is the main glutamate transporter in *S. aureus*.

### 2.6 Ammonium and glutamine but not glutamate stimulate the growth of *S. aureus* in defined medium containing glucose as carbon source

**Figure 6:**
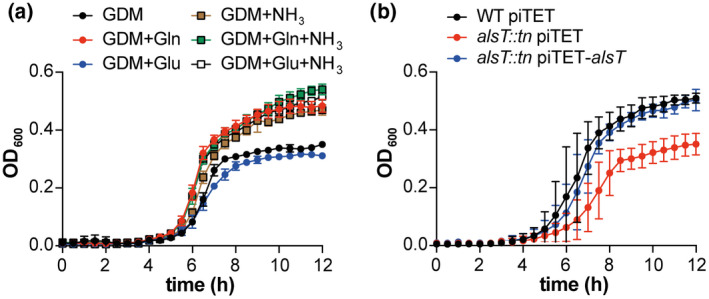
Addition of glutamine and ammonium but not glutamate stimulates the growth of *S. aureus* in glucose‐containing defined medium. (a) Bacterial growth curves. The WT *S. aureus* strain LAC* was grown in 96‐well plates in glucose‐containing defined medium (GDM) or in GDM supplemented with glutamine (Gln), glutamate (Glu), ammonium (NH_3_) or combinations thereof as specified in the legend. OD_600_ readings were determined every 30 min and the average and SDs of three biological replicates plotted. (b) Bacterial growth curves. *S. aureus* strains LAC* piTET (WT piTET), the *alsT* mutant strain LAC*alsT::tn piTET and the complementation strain LAC*alsT::tn piTET‐*alsT* were grown in GDM + Gln medium supplemented with 200 ng/µl Atet. OD_600_ readings were determined every 30 min and the average and SDs of three biological replicates plotted

Glutamine and glutamate are important amino acids that can serve, together with ammonium, as nitrogen sources for the synthesis of many other cellular metabolites. Since *S. aureus* is phenotypically auxotroph for many amino acids and at the same time can use several amino acids as carbon and nitrogen sources, it is not possible to grow this organism in any of the typical minimal media that are used to assess the ability of bacteria to specifically use ammonium, glutamine or glutamate as nitrogen sources. However, to begin to examine the effect of these compounds on the growth of *S. aureus*, growth curves were performed with the WT LAC* strain in glucose containing defined medium (GDM), containing essential vitamins, metals and 17 amino acids but lacking ammonium, glutamine and glutamate as potential nitrogen/amino acid sources (see Table S1 for medium composition). In addition, the WT LAC* strain was also grown in GDM containing glutamine (GDM + Gln), glutamate (GDM + Glu), ammonium (GDM + NH_3_), glutamine and ammonium (GDM + Gln + NH_3_) or glutamate and ammonium (GDM + Glu+NH_3_). The addition of glutamine or ammonium alone or in combinations stimulated the growth of the WT LAC* strain as compared to its growth in GDM (Figure 6a). On the other hand, no growth improvement was seen in the presence of glutamate (GDM + Glu) (Figure 6a). To examine the contribution of the glutamine and glutamate transporters AlsT and GltS, additional growth curves were performed in the different media with WT LAC* as well as strains LAC*alsT::tn and LAC*gltS::tn (Figure S4). Similar growth profiles were observed for all strains in the different media (Figure S4), except in GDM + Gln, in which the *alsT* mutant strain exhibited reduced growth compared to the WT and *gltS* mutant strains (Figure S4b). The growth defect could be restored in the *alsT* complementation strain harbouring plasmid piTET‐*alsT* (Figure 6b). Taken together, these data indicate that ammonium and glutamine are preferred over glutamate for the growth of *S. aureus*. The observation that the addition of ammonium improves the growth of *S. aureus* indicates that our base medium is likely nitrogen limiting and suggests that glutamine but not glutamate can likely also serve as nitrogen source under these growth conditions. Finally, these data further confirm the importance of AlsT for glutamine uptake in *S. aureus.*

### 2.7 Ammonium and glutamine uptake lead to a reduction in c‐di‐AMP levels in *S. aureus*

**Figure 7:**
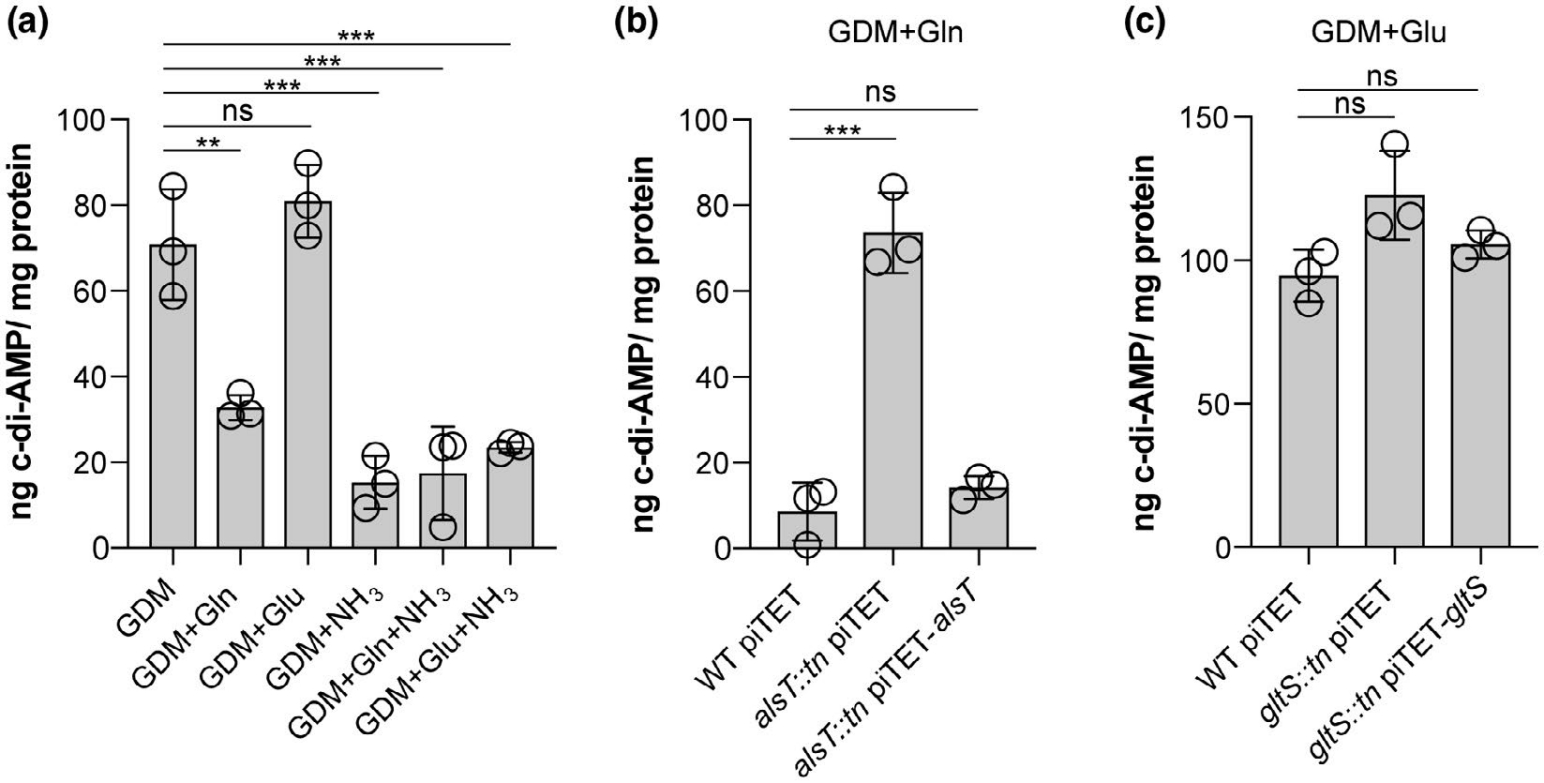
Ammonium and glutamine uptake inhibit c‐di‐AMP production in *S. aureus*. (a–c) Cellular c‐di‐AMP levels. (a) The WT *S. aureus* strain LAC* was grown in GDM or GDM supplemented with glutamine (Gln), glutamate (Glu), ammonium (NH_3_), or combinations thereof as indicated on the *x* axis. Cell extracts were prepared, and c‐di‐AMP concentrations measured using a competitive ELISA assay. The average values and SDs from three biological replicates were plotted as ng c‐di‐AMP/mg protein. For statistical analysis, one‐way ANOVAs followed by Dunnett's multiple comparison tests were performed to identify statistically significant differences between the different media as compared to GDM (ns = not significant, ** = *p* < .001, *** = *p* < .0001). (b) *S. aureus* strains LAC* piTET (WT piTET), LAC*alsT::tn piTET and the complementation strain LAC*alsT::tn piTET‐*alsT* were grown in GDM + Gln supplemented with 200 ng/µl Atet and c‐di‐AMP levels determined and plotted as described in (a). (c) *S. aureus* strains LAC* piTET (WT piTET), LAC*gltS::tn piTET and the complementation strain LAC*gltS::tn piTET‐*gltS* were grown in GDM + Glu supplemented with 200 ng/µl Atet and c‐di‐AMP levels determined and plotted as described in (a)

For *B. subtilis,* it has been reported that the addition of glutamine, glutamate or ammonium to a defined growth medium can affect cellular c‐di‐AMP levels (Gundlach et al., 2015). It was further proposed that glutamate uptake and to some extent also ammonium uptake leads to an activation of c‐di‐AMP synthesis in this organism (Gundlach et al., 2015). To assess if the presence of glutamine, glutamate or ammonium would also affect c‐di‐AMP levels in *S. aureus*, the intracellular c‐di‐AMP concentrations were determined for the WT *S. aureus* strain LAC* following growth in GDM, GDM + Gln, GDM + Glu, GDM + NH_3_, GDM + Gln+NH_3_ and GDM + Glu+NH_3_. Using a competitive ELISA assay, c‐di‐AMP could be readily detected in bacteria grown in GDM, our base medium (Figure 7a). Similar amounts of c‐di‐AMP were detected in bacteria grown in the glutamate‐containing medium (GDM + Glu); however, the c‐di‐AMP levels were significantly lower in bacteria grown in medium containing either ammonium or glutamine (GDM + Gln, GDM + NH_3_, GDM + Gln+NH_3_, GDM + Glu+NH_3_) (Figure 7a). To verify that the addition of glutamine reduces c‐di‐AMP production and to investigate the contribution of the glutamine transporter AlsT to this inhibition, c‐di‐AMP levels were determined for WT piTET, the *alsT* mutant LAC*alsT::tn piTET and the complementation strain LAC*alsT::tn piTET‐*alsT* following growth in the glutamine containing medium GDM + Gln. While again low c‐di‐AMP levels were detected for the WT strain, the c‐di‐AMP levels increased significantly in the *alsT* mutant and were restored back to WT levels in the complementation strain (Figure 7b). A similar experiment was performed with the *gltS* mutant and complementation strain in the glutamate containing medium GDM + Glu. High and similar c‐di‐AMP levels were detected for all strains (Figure 7c), indicating that neither the addition of glutamate to the medium nor its uptake impacts c‐di‐AMP production in *S. aureus* under our test conditions. Taken together, these data highlight that ammonium as well as AlsT‐mediated glutamine uptake, which likely results in a higher intracellular glutamine concentration, represses c‐di‐AMP production in *S. aureus*.

### 2.8 The inhibition of the c‐di‐AMP production by glutamine and ammonium is not mediated by GdpP or YbbR

**Figure 8:**
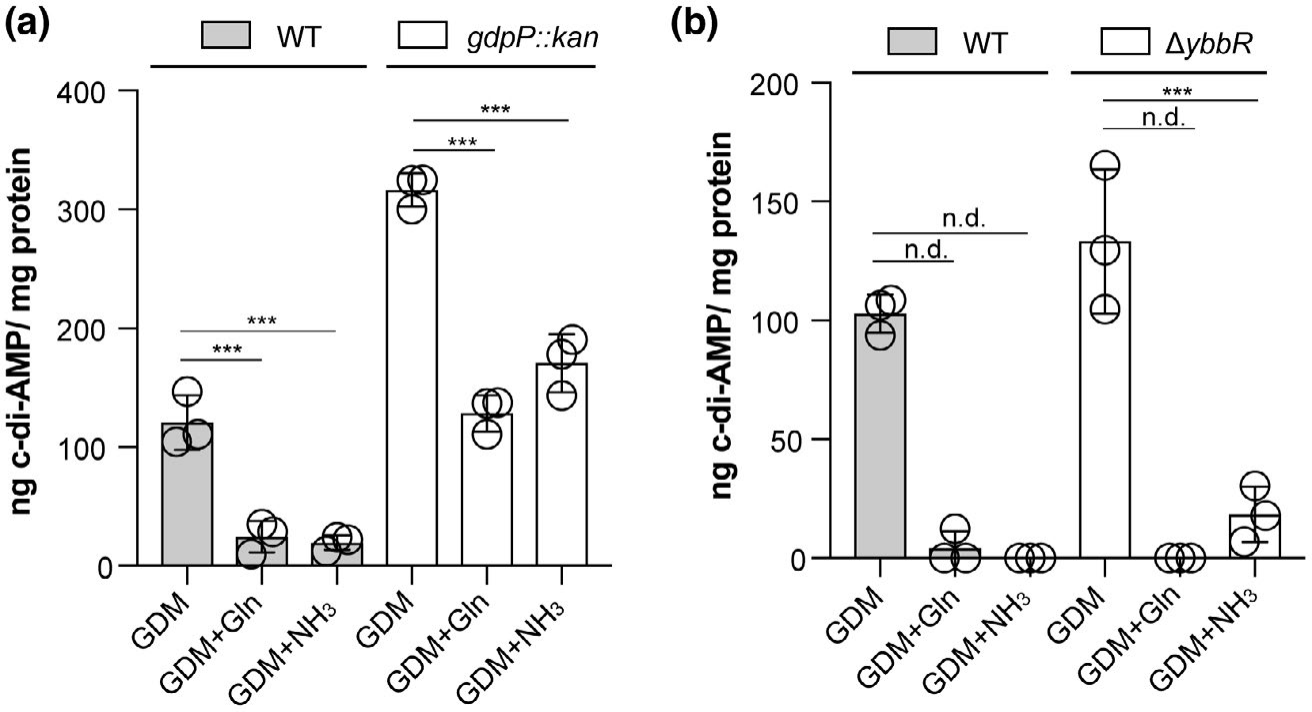
The inhibition of c‐di‐AMP production by glutamine and ammonium is independent of GdpP and YbbR. (a, b) Cellular c‐di‐AMP levels. (a) *S. aureus* strains LAC* (WT) and LAC*gdpP::kan (*gdpP::kan)* were grown in GDM or in GDM containing glutamine (GDM + Gln) or ammonium (GDM + NH_3_). Cell extracts were prepared, and c‐di‐AMP concentrations measured using a competitive ELISA assay. The average values and SDs from three biological replicates were plotted as ng c‐di‐AMP/ mg protein. For statistical analysis one‐way ANOVAs followed by Dunnett's multiple comparison tests were performed to identify statistically significant differences between the different media as compared to the GDM medium (n.d. = not determined. Statistical analysis was not performed due to values being below our detection limit; these values were set to 0, ** = *p* < .001, *** = *p* < .0001). (b) Same as in (a) but using *S. aureus* strains LAC* (WT) and LAC*Δ*ybbR* (Δ*ybbR*)

The observed reduction of c‐di‐AMP levels in the presence of glutamine or ammonium could potentially be achieved through an increase in the activity of the c‐di‐AMP‐specific phosphodiesterase GdpP. To investigate this, cellular c‐di‐AMP levels were compared between WT LAC* and the isogenic *gdpP* mutant strain LAC*gdpP::kan. As previously reported for strain LAC*gdpP::kan following growth in TSB medium (Corrigan et al., 2011), the c‐di‐AMP levels were also increased in the *gdpP* mutant compared to the WT strain following growth in GDM, the glucose containing defined medium used as part of this study (Figure 8a). However, a significant reduction in the cellular c‐di‐AMP levels was also seen for the *gdpP* mutant following the addition of glutamine or ammonium to the medium (Figure 8a). This indicates that the reduction in c‐di‐AMP levels upon addition of glutamine or ammonium is likely due to decreased synthesis by DacA and not increased degradation by GdpP. We next tested the involvement of YbbR, a proposed c‐di‐AMP cyclase regulator, by comparing the cellular c‐di‐AMP levels produced by WT LAC* and strain LAC*Δ*ybbR*. Similar c‐di‐AMP levels were detected in the WT and *ybbR* mutant in GDM medium (Figure 8b). The addition of glutamine or ammonium to the medium led also to a large reduction in the cellular c‐di‐AMP in the *ybbR* mutant strain (Figure 8b). These data suggest that the observed reduction of c‐di‐AMP production in the presence of glutamine and ammonium is neither mediated by GdpP nor YbbR, and hence involves a different regulator protein, or that the cellular glutamine and nitrogen levels are directly sensed by the cyclase DacA.

## 3 DISCUSSION

Over the last decade, considerable evidence has emerged that c‐di‐AMP plays a major role in osmotic regulation in bacteria, primarily by positively regulating potassium export or negatively regulating potassium and osmolyte uptake (Chin et al., 2015; Corrigan et al., 2013; Devaux et al., 2018; Gundlach, Commichau, et al., 2017; Gundlach, Herzberg, Hertel, et al., 2017; Gundlach, Herzberg, Kaever, et al., 2017c; Gundlach et al., 2019; Huynh et al., 2016; Kim et al., 2015; Moscoso et al., 2015; Pham et al., 2018; Pham & Turner, 2019; Quintana et al., 2019; Rocha et al., 2019; Schuster et al., 2016; Zarrella et al., 2018). However, individual c‐di‐AMP target proteins identified thus far are themselves not essential. Therefore, the essentiality of c‐di‐AMP is likely due to its ability to regulate multiple target proteins simultaneously. Furthermore, in the absence of this molecule, many transporters are activated rather than inactivated, likely leading to accumulation of toxic levels of metabolites, such as potassium and osmolytes. Consistent with this idea, inactivating mutations in potassium uptake systems, oligopeptide and osmolyte transporters have been reported to rescue the growth defect of bacteria unable to produce c‐di‐AMP (Devaux et al., 2018; Gundlach, Herzberg, Hertel, et al., 2017; Gundlach, Herzberg, Kaever, et al., 2017c; Pham et al., 2018; Whiteley et al., 2017, 2015; Zeden et al., 2018). We have previously shown that inactivation of the main glycine betaine transporter OpuD bypasses the requirement of c‐di‐AMP for the growth of *S. aureus* in rich medium (Zeden et al., 2018). We hypothesize that inactivation of OpuD might help a c‐di‐AMP null strain survive by allowing bacteria to re‐establish their osmotic balance. Bacteria of the *dacA/opuD* mutant strain, which cannot produce c‐di‐AMP but are also defective in glycine betaine transport, are similar in size to WT bacteria (Figure 1). At this point, it is not known if c‐di‐AMP can directly bind to and regulate the activity of the *S. aureus* OpuD protein. We attempted to address this question; however, despite using multiple different approaches, we were unable to produce sufficient amounts of the full‐length OpuD membrane protein to perform protein/nucleotide interaction studies. On the other hand, a direct role for c‐di‐AMP in the control of glycine betaine or betaine transporters has been reported in *S. agalactiae* and *L. lactis*. In these organisms, c‐di‐AMP binds to the transcriptional regulator BusR, which controls the expression of the predicted glycine betaine or betaine transporter BusAB (Devaux et al., 2018; Pham et al., 2018).

Bacteria of the *dacA/alsT* suppressor strain, which survive in the absence of c‐di‐AMP, remained enlarged, indicating that the essentiality of c‐di‐AMP is bypassed in this strain potentially through a different mechanism. Here, we show that AlsT is an efficient glutamine transporter in *S. aureus* (Figure 2). These findings indicate that eliminating or reducing the ability of *S. aureus* to take up glutamine from rich growth medium rescues the growth of an *S. aureus* unable to produce c‐di‐AMP. There are several (not mutually exclusive) possibilities how preventing glutamine uptake could rescue the growth of a c‐di‐AMP null strain in rich medium. Glutamine as well as proline have been shown to accumulate in *S. aureus* under NaCl stress conditions (Anderson & Witter, 1982). While it has been suggested that the glutamine accumulation is due to synthesis rather than uptake (Anderson & Witter, 1982), these data highlight that glutamine likely plays an important role in osmotic regulation in *S. aureus.* Despite the cell size not being restored in the *dacA/alsT* suppressor strain, blocking glutamine uptake could potentially still help bacteria to better balance their cellular osmolality during growth in rich medium in the absence of c‐di‐AMP. Another possible explanation how eliminating glutamine uptake could allow *S. aureus* to grow in the absence of c‐di‐AMP could be connected to changes in metabolism and TCA cycle activity. In *L. monocytogenes,* an increased flux of pyruvate into the TCA cycle has been described for a strain unable to produce c‐di‐AMP (Sureka et al., 2014). As a consequence, an accumulation of citrate and increased carbon flux into glutamine/glutamate was observed, which resulted in a metabolic imbalance and growth defect (Sureka et al., 2014). Perhaps similar to the observations in *L. monocytogenes*, the absence of c‐di‐AMP could also boost TCA cycle activity in *S. aureus*, thus leading to glutamine accumulation and a metabolic imbalance. Hence, the lack of c‐di‐AMP combined with active glutamine uptake could fuel the bacterial metabolism and the resulting metabolic imbalance might become toxic to the cell, similar as observed for *L. monocytogenes* (Sureka et al., 2014; Whiteley et al., 2017).

In a recent study investigating genetic determinants required for eDNA release during biofilm formation, it was found that inactivation of GdpP as well as of AlsT, resulted in a significant decrease in eDNA release and in an increase in resistance to Congo red (DeFrancesco et al., 2017). Therefore, inactivation of AlsT and preventing/reducing glutamine uptake might lead to alterations in the bacterial cell wall that make bacteria more resistant to cell lysis. Such changes could also be an advantage during osmotic stress or c‐di‐AMP deficiency. Indeed, we have recently shown a correlation between specific changes in the peptidoglycan structure and the NaCl stress resistance in *S. aureus* (Schuster et al., 2019). In addition, since the cellular c‐di‐AMP levels are significantly higher in the *gdpP* as well as the *alsT* mutant strains compared to WT (Figures 7b and 8a), the underlying mechanistic bases for the decrease in eDNA release observed for the *gdpP* and *alsT* mutant strains might be related.

The actual stimuli and underlying molecular mechanisms that regulate c‐di‐AMP production in bacterial cells are at the moment poorly understood. As part of this study, we show that ammonium and AlsT‐mediated glutamine uptake (and likely a reduction in the cellular glutamine levels) but not GltS‐mediated glutamate uptake negatively impacts c‐di‐AMP production (Figure 7). Changes in cellular c‐di‐AMP levels depending on the presence of ammonium, glutamine or glutamate have already been reported for *B. subtilis* (Gundlach et al., 2015). For *B. subtilis* it has been suggested that glutamine and to some extent ammonium uptake stimulates c‐di‐AMP production (Gundlach et al., 2015). Here, we show that in *S. aureus* ammonium and glutamine uptake leads to an inhibition of c‐di‐AMP production rather than glutamate promoting its synthesis (Figure 7). The decrease in c‐di‐AMP production in the presence of ammonium or glutamine is likely achieved by reducing the activity of the c‐di‐AMP cyclase DacA and not by activation of the c‐di‐AMP‐specific phosphodiesterase GdpP. This conclusion is based on our observation that the cellular c‐di‐AMP levels are also decreased in a *gdpP* mutant strain upon addition of glutamine or ammonium (Figure 8a). Current evidence suggests that the activity of DacA can be regulated through the interaction with two proteins: the membrane anchored and proposed DacA regulator protein YbbR (also named as CdaR in other bacteria) and the phosphoglucosamine mutase enzyme GlmM (Gundlach et al., 2015; Pham, Liang, Marcellin, & Turner, 2016; Tosi et al., 2019; Zhu et al., 2016). We could exclude that the observed reduction in cellular c‐di‐AMP levels in the presence of ammonium or glutamine is mediated by YbbR, as a *ybbR* mutant showed a similar decrease in the c‐di‐AMP levels as observed for the WT strain (Figure 8b). GlmM has been shown to be a negative regulator of DacA activity both in vivo and in vitro (Pham et al., 2018; Tosi et al., 2019)*.* However, since GlmM is likely an essential enzyme in *S. aureus*, we were unable to construct a *glmM* mutant and test its involvement in the observed repression of c‐di‐AMP synthesis in the presence of ammonium or glutamine as we did for the *gdpP* and *ybbR* mutant strains. Nevertheless, with this work, we not only identified main glutamine and glutamate transporters in *S. aureus,* but we also linked the c‐di‐AMP signalling network to central nitrogen metabolism in *S. aureus.* It will be interesting to determine in future studies the mechanistic bases for the observed changes in cellular c‐di‐AMP levels depending on ammonium and glutamine uptake and the involvement of GlmM or other factors in this process.

## 4 EXPERIMENTAL PROCEDURES

### 4.1 Bacterial strains and culture conditions

Bacterial strains used in this study are listed in Table 1. *S. aureus* strains were grown in tryptic soy broth (TSB), where indicated supplemented with 0.4 M NaCl, tryptic soy agar (TSA) or glucose defined medium (GDM). GDM was prepared similar to the chemically defined medium (CDM) reported in an earlier study (Zeden et al., 2018), with some modifications. The detailed content of the GDM (which contains essential vitamins, trace metals, amino acids and glucose as a carbon source but lacks ammonium, glutamine or glutamate as a potential nitrogen or amino acid source) is shown in Table S1. In addition to the GDM, GDM containing glutamine (GDM + Gln), glutamate (GDM + Glu), ammonium (GDM + NH_3_), glutamine and ammonium (GDM + Gln+NH_3_) or glutamate and ammonium (GDM + Glu+NH_3_) were used as part of this study (for exact composition see Table S1). *Escherichia coli* strains were grown in lysogeny broth (LB). Where appropriate, antibiotics and/or inducers were added to the media at the following concentration: 200 ng/ml anhydrotetracycline (Atet), 90 µg/ml Kanamycin (Kan), 10 µg/ml Erythromycin (Erm), 7.5 or 10 µg/ml Chloramphenicol (Cam) and Ampicillin (Amp) 100 µg/ml.

**Table 1:** Bacterial strains used in this study

Unique ID	Strain name and resistance	Source
	*Escherichia coli* strains	
ANG284	XL1‐Blue piTET; AmpR	Gründling and Schneewind (2007)
ANG2154	DH10B pIMAY; CamR	(Monk, Shah, Xu, Tan, & Foster, 2012)
ANG3724	IM08B	Monk, Tree, Howden, Stinear, and Foster (2015)
ANG3928	IM08B piTET; AmpR	Zeden et al. (2018)
ANG3937	XL1‐Blue piTET‐*alsT*; AmpR	This study
ANG3955	IM08B piTET‐*alsT*; AmpR	This study
ANG5494	XL1‐Blue piTET‐*gltS*; AmpR	This study
ANG5495	IM08B piTET‐*gltS;* AmpR	This study
	*Staphylococcus aureus strains*	
AH1263	LAC* Erm sensitive CA‐MRSA USA300 strain (ANG1575)	Boles, Thoendel, Roth, and Horswill (2010)
ANG1961	LAC*gdpP::kan; KanR	Corrigan et al. (2011)
ANG3301	LAC*Δ*ybbR*	Bowman et al. (2016)
ANG3664	LAC*dacA_G206S_; KanR	Bowman et al. (2016)
ANG3666	LAC*dacA::kan (*dacA*) KanR	Zeden et al. (2018)
ANG3835	LAC*dacA::kan‐S7 (LAC*dacA/opuD); KanR	Zeden et al. (2018)
ANG3838	LAC*dacA::kan‐S10 (LAC*dacA/alsT)]; KanR	Zeden et al. (2018)
ANG3940	NE142 (*alsT::tn*)—NMTN strain	Fey et al. (2013)
ANG4054	LAC* piTET; CamR	Zeden et al. (2018)
ANG4803	LAC*alsT::tn; ErmR	This study
ANG4854	LAC*alsT::tn piTET*‐alsT;* ErmR CamR	This study
ANG4853	LAC*alsT::tn piTE*;* ErmR CamR	This study
ANG4968	NE1463 (JE2 SAUSA300_*0914::tn*)—NMTN strain	Fey et al. (2013)
ANG5070	NE153 (JE2 *glnQ::tn*)—NMTN strain	Fey et al. (2013)
ANG5141	LAC*0914::tn; ErmR	This study
ANG5242	LAC*glnQ::tn; ErmR	This study
ANG5309	NE566 (JE2 *gltT::tn*)—NMTN strain	Fey et al. (2013)
ANG5310	NE560 (JE2 *gltS::tn*)—NMTN strain	Fey et al. (2013)
ANG5366	LAC*gltT::tn; ErmR	This study
ANG5367	LAC*gltS::tn; ErmR	This study
ANG5492	LAC*gltS::tn piTET; ErmR CamR	This study
ANG5493	LAC*gltS::tn piTET‐*gltS*; ErmR CamR	This study

### 4.2 Bacterial strain construction

All strains used in this study are listed in Table 1 and primers used in this study are listed in Table 2. The transposon insertion sites in the Nebraska transposon mutant library (NTML) strains (Fey et al., 2013) used as part of this study were confirmed by PCR and sequencing. The transposon and surrounding regions were moved by phage transduction using phage 85 into the *S. aureus* LAC* strain background. This resulted in the generation of *S. aureus* strains LAC*alsT::tn (*SAUSA300_1252::tn*; ANG4803), LAC*0914::tn (*SAUSA300_0914::tn*; ANG5141), LAC*glnQ::tn (*SAUSA300_1807::tn;* ANG5070), LAC*gltT::tn (*SAUSA300_2329::tn*; ANG5366) and LAC*gltS::tn (*SAUSA300_2291::tn*; ANG5367). The transposon insertion in the respective gene was again confirmed by PCR and sequencing. For complementation analysis, the Atet inducible single‐copy integration plasmids piTET‐*alsT* and piTET‐*gltS* were constructed. To this end, *alsT* (*SAUSA300_1252*) and *gltS* (*SAUSA300_2291*) were amplified using LAC* chromosomal DNA and primers ANG2250/ANG2251 and ANG3209/ANG3210 respectively. The products as well as piTET were digested with AvrII and SacII and then ligated. Plasmid piTET‐*alsT* was recovered in *E. coli* strain XL1‐Blue (yielding strain ANG3937), shuttled through *E. coli* strain IM08B (strain ANG3955) and then introduced into LAC*alsT::tn (ANG4803), yielding strain LAC*alsT::tn piTET*‐alsT* (ANG4854). As a control, plasmid piTET was also introduced into LAC*alsT::tn (ANG4803) yielding strain LAC*alsT::tn piTET (ANG4853). Plasmid piTET‐*gltS* was transformed into *E. coli* XL1‐Blue (yielding strain ANG5494), shuttled through *E. coli* IM08B (yielding strain ANG5495) and transformed into LAC*gltS::tn, yielding the complement strain LAC*gltS::tn piTET‐*gltS* (ANG5493). As a control, the piTET plasmid was transformed into LAC*gltS::tn strain, yielding the strain LAC*gltS:tn piTET (ANG5492). Correct plasmid integration into the *geh* locus was confirmed by PCR and the sequences of all plasmid inserts were confirmed by fluorescent automated sequencing.

**Table 2:** Cloning primers used in this study

Primer ID	Name	Sequence
ANG2250	5‐AvrII‐*alsT*	AGTCCCTAGGCGGTCTAATTTTATAGAAGG
ANG2251	3‐SacII‐*alsT*	TCCCCGCGGGGTTTATTTGATTTTTATATAATGAATCG
ANG3209	5‐AvrII‐*gltS*	ATACCTAGGAGGGAGAGGGATATTCAACAAGGGGGATTTG
ANG3210	3‐SacII‐*gltS*	GCCCGCGGTTTAACTAAACCATTGTATGAATCCCATAATG

### 4.3 Bacterial growth curves and amino acid analysis in culture supernatants

*S. aureus* strains LAC* and LAC*alsT::tn were grown overnight in TSB supplemented with 10 µg/ml erythromycin where appropriate. Overnight cultures were then diluted to an OD_600_ of 0.01 into 50 ml of fresh TSB. Cultures were incubated at 37°C with aeration, and OD_600_ values determined every hour. The experiment was performed with three biological replicates and the average OD_600_ values and standard deviations (SDs) were plotted. Using the same cultures, supernatant samples were prepared at the 0, 6, 10 and 12 hr time points and amino acid levels determined as previously described using an amino acid analyser (Halsey et al., 2017). For measuring the growth of *S. aureus* strains LAC*, LAC* piTET, LAC*alsT::tn, LAC*alsT::tn piTET, LAC*alsT::tn piTET‐*alsT* and LAC*gltS::tn in GDM, GDM + Gln, GDM + Glu, GDM + NH_3_, GDM + Gln+NH_3_ or GDM + Glu+NH_3_, the bacteria were grown overnight in TSB medium supplemented with chloramphenicol and erythromycin where appropriate. Next day, bacteria from a 1 ml aliquot were washed twice with PBS and diluted to an OD_600_ of 0.005 in the indicated GDM. LAC* WT, LAC*alsT::tn and LAC*gltS::tn were grown in GDM, GDM + Gln, GDM + Glu, GDM + NH_3_, GDM + Gln+NH_3_ and GDM + Glu+NH_3_, while LAC* piTET, LAC*alsT::tn piTET and LAC*alsT::tn piTET‐*alsT* were grown in GDM + Gln supplemented with 200 ng/ml Atet. One hundred microlitres of the diluted cultures (six technical replicates) were transferred into wells of a 96‐well plate and the plate was then incubated with shaking (500 rpm) in a plate reader and OD_600_ readings determined every 30 min. The average values of the technical replicates were determined for each strain. The experiment was performed three times and the average readings and standard deviations were plotted.

### 4.4 γ‐L‐glutamyl hydrazide susceptibility assay

The susceptibility of *S. aureus* LAC*, LAC*alsT::tn (ANG4803), LAC*0914::tn (ANG5141) and LAC*glnQ::tn (ANG5242) to the toxic glutamine analogue γ‐L‐glutamyl hydrazide (Alfa Aesar, MA, USA) was determined using a similar method as previously reported (Zhu et al., 2009). Briefly, the different strains were grown overnight at 37°C in 5 ml TSB medium, supplemented with 10 μg/ml erythromycin where appropriate. Next day, the bacteria were washed twice with PBS, diluted to an OD_600_ of 0.005 in GDM + NH_3_. Next, 10 μl of water (0 mM control) or 10 μl of a γ‐L‐glutamyl hydrazide solution dissolved in water was added to 0.99 ml aliquots of these bacterial suspension to give a final concentration of 20, 40, 60 or 80 μg/ml, respectively. One hundred microlitres were subsequently transferred in four replicates into wells of a 96‐well plates and the plate incubated at 37°C with shaking (500 rpm) in a plate reader and OD_600_ readings determined every 10 min for 12 hr. The experiment was performed three times and the average OD_600_ values of the three experiments presented as growth curves. The average values and SDs of the OD_600_ values from the 7 hr time point were also plotted against the different γ‐L‐glutamyl hydrazide concentrations.

### 4.5 Microscopic analysis and cell size measurements

The microscopic analysis to determine bacterial cell sizes was performed essentially as previously described (Zeden et al., 2018). Briefly, *S. aureus* strains LAC*, LAC*dacA::kan, LAC*dacA_G206S_, LAC*dacA/opuD (ANG3835) and LAC*dacA/alsT (ANG3838) were grown overnight at 37°C in TSB or TSB supplemented with 0.4 M NaCl where stated. Next day, the cultures were diluted to an OD_600_ of 0.01 and grown for 3 hr at 37°C to mid‐exponential phase (OD_600_ of 0.5–0.9). One hundred microlitres of these cultures were then stained for 20 min at 37°C with Vancomycin‐BODIPY FL used at a final concentration of 2 µg/ml. One and a half microlitres of each sample were spotted onto a thin 1.5% agarose gel patch prepared in H_2_O or in 0.4 M NaCl and the bacteria subsequently imaged at 1,000× magnification using an Axio Imager A2 Zeiss microscope equipped with a GFP filter set. Images were acquired using the ZEN 2012 (blue edition) software. The bacterial cell diameters were determined using the Fiji software. Only nondividing cells (cells without any obvious fluorescent dots or lines at the mid‐cell), were used for cell diameter measurements. The cell diameters of 50 cells were measured and the average cell diameter determined. The experiment was conducted three or four times (as indicated in the figure legend) and the averages and standard deviations of the average cell diameters plotted.

### 4.6 Uptake assays using ^14^C‐labelled amino acids

Uptake assays were conducted as previously described with some minor modifications (Zeden et al., 2018). Briefly, *S. aureus* strains were streaked on TSA or TSA 0. 4M NaCl plates with appropriate antibiotics and the plates incubated overnight at 37°C. Bacteria were subsequently scraped off from the plates and suspended in 1 ml PBS pH 7.4 buffer and the OD_600_ determined. Fifty ml of GDM + Glu+NH_3_ (where indicated with 200 ng/ml of the inducer Atet added) were inoculated with the appropriate bacterial suspensions to an OD_600_ of 0.05. The cultures were grown at 37°C to an OD_600_ between 0.4 and 0.9 and bacteria from an OD_600_ equivalent of 8 were harvested by centrifugation for 10 min at 19,000 x g at RT. Supernatants were discarded and the bacterial pellets were suspended in 2 ml of GDM + NH_3_. The OD_600_ of the cell suspensions were measured and the cells diluted to an OD_600_ of approximately 1. The OD_600_ was re‐measured and this measurement used for normalization purposes. Five hundred and fifty microlitres of these cell suspensions were aliquoted into 50 ml conical tubes and 100 µl used to measure the background radiation, by filtering the cells onto a nitrocellulose membrane filter, followed by a wash step with 16 ml PBS. Then, 6.2 µl of glutamine, L‐[14C(U)] (Hartmann Analytic, MC1124) or glutamic acid, L‐[14C(U)] (Hartmann Analytic, MC156) was added to the remaining 450 µl sample. One hundred microlitres aliquots were filtered 0, 3, 6 and 9 min after addition of the radiolabelled amino acid and the filters were then washed two times with 16 ml of PBS pH 7.4. The filters were subsequently dissolved in 9 ml of Filter Count scintillation cocktail (Perkin Elmer) and the radioactivity measured in counts per minute (CPM) using a Wallac 1,409 DSA liquid scintillation counter. The CPMs were then normalized to the OD_600_ reading of the final cell suspension and the means and standard deviations of the CPM/ml OD_600_ = 1 of three or four (as indicated in the figure legends) independent experiments were plotted.

### 4.7 Determination of cellular c‐di‐AMP levels by competitive ELISA

Intracellular c‐di‐AMP levels in WT LAC* and the indicated *S. aureus* mutant strains were determined using a previously described competitive ELISA method (Underwood, Zhang, Metzger, & Bai, 2014) and a slightly modified method for the preparation of *S. aureus* samples (Bowman, Zeden, Schuster, Kaever, & Gründling, 2016). Briefly, single colonies of the WT LAC* strain were picked from TSA plates and used to inoculate 5 ml of GDM, GDM + Gln, GDM + Glu, GDM + NH_3_, GDM + Gln+NH_3_ and GDM + Glu+NH_3_. Colonies of the strains LAC*gdpP::kan and LAC*Δ*ybbR* were inoculated into 5 ml of GDM, GDM + Glu and GDM + Gln. Colonies of strains LAC* piTET, LAC*alsT::tn piTET and LAC*alsT::tn piTET‐*alsT* were inoculated into GDM + Gln containing 200 ng/ml Atet and colonies of strains LAC* piTET, LAC*gltS::tn piTET and LAC*gltS::tn piTET‐*gltS* were inoculated into GDM + Glu supplemented with 200 ng/ml Atet. All cultures were incubated for 18 hr at 37°C with shaking. Next, bacteria from 4.5 ml culture were collected by centrifugation, washed three times with PBS and subsequently suspended in 0.75 to 1 ml 50 mM Tris pH 8 buffer supplemented with 20 ng/ml lysostaphin and the cells were lysed by bead beating. The lysates were cleared by centrifugation for 5 min at 17,000× *g* and the supernatant transferred to a new tube. A small sample aliquot was removed, and the protein concentration determined for normalization purposes using a Pierce BCA protein assay kit (Thermo Scientific, Waltham, MA, USA). The remainder of the sample was heated to 95°C for 10 min. For the competitive ELISA assay, the samples were diluted to a protein concentration of 100, 200, 400 or 500 μg/ml as, appropriate. ELISA plates were prepared by adding 100 μl of coating buffer (50 mM Na_2_C0_3_, 50 mM NaHCO_3_, pH 9.6) containing 10 μg/ml of the c‐di‐AMP binding protein CpaA_SP_ to each well of a 96 well NUNC MaxiSorp plate (Thermo Scientific, Waltham, MA, USA) and the plate was incubated for approximately 18 hr at 4°C. Next, the plate was washed three times with 200 μl PBST pH 7.4 (10 mM Na_2_HPO_4_, 1.8 mM KH_2_PO_4_ 137 mM NaCl, 2.7 mM KCl, 0.05% (v/v) Tween 20), blocked for 1 hr at 18°C with 150 μl blocking solution (1% BSA in PBS pH 7.4) and washed three times with 200 μl PBST. Fifty microlitres of the samples (three biological replicates and three technical replicates) or standards (two technical replicates) were mixed with 50 μl of a 50 nM biotinylated c‐di‐AMP solution prepared in 50 mM Tris pH 8 buffer. For the standard curve, c‐di‐AMP standards were prepared in 50 mM Tris pH 8 buffer at concentrations of 0, 12.5, 25, 37.5, 50, 75, 100 and 200 nM. Following the addition of the samples and the standards, the plate was incubated for 2 hr at 18°C and then washed three times with PBST. Next, 100 μl of a high‐sensitivity streptavidin‐HRP solution (Thermo Scientific, Waltham, MA, USA) diluted 1:500 in PBS was added to each well and the plate was incubated for 1 hr at 18°C. The plate was washed again 3 x with 200 μl PBST and 100 μl of a developing solution (0.103 M NaHPO_4_, 0.0485 M citric acid, 500 mg/L o‐phenylenediamine dihydrochloride, 0.03% H_2_O_2_) was added to each well and the plate incubated for 15 min at 18°C. The reaction was then stopped by adding 100 μl of 2 M H_2_SO_4_ solution. The absorbance was measured in a plate reader at a wavelength of 490 nm and c‐di‐AMP concentrations were calculated as ng c‐di‐AMP/ mg protein.

## CONFLICT OF INTEREST

The authors have no conflicts of interest to declare.

## AUTHOR CONTRIBUTIONS

MSZ, IK and AG designed the study; MSZ, IK and CFS acquired the data; MSZ, IK, CFS, VCT, PDF and AG designed experiments, analysed and interpreted the data; MSZ, IK and AG prepared the figures and wrote the original draft of the manuscript. All authors approved the final version of the manuscript.

## Data Availability

The data supporting the findings of this study will be openly available.
