# Supplementary material for: Identification of the main glutamine and glutamate transporters in Staphylococcus aureus and their impact on c‐di‐AMP production
Source: Mol Microbiol. 2020 Feb 11;113(6):1085–100. doi: 10.1111/mmi.14479 (PMC7299772; doi:10.1111/mmi.14479)
Supplement: Supplementary file 1 [file MMI-113-1085-s001.pdf]

Supplementary material for manuscript

Identification of the main glutamine and glutamate transporters in *Staphylococcus aureus* and their impact on c-di-AMP production

Merve S. Zeden[a^], Igor Kviatkovski[a^], Christopher F. Schuster[a], Vinai C. Thomas[b], Paul D. Fey[b] and Angelika Gründling[a#]

[a] Section of Molecular Microbiology and Medical Research Council Centre for Molecular Bacteriology and Infection, Imperial College London, London SW7 2AZ.

[b] University of Nebraska Medical Center, Department of Pathology and Microbiology, Omaha, Nebraska, USA.

^ These authors contributed equally

[#] To whom correspondence should be addressed: Angelika Gründling – a.grundling@imperial.ac.uk

Content:

Table S1. Composition of the glucose defined media (GDM) used in this study

Fig. S1: Growth and amino acid consumption analysis to determine the function of AlsT.

Fig. S2. CLUSTAL Omega alignment of *B. subtilis* and *S. aureus* AlsT/GlnT homologs.

Fig. S3. LAC**alsT::tn* shows increased resistance to the toxic glutamine analogue γ-L-glutamyl hydrazide.

Fig. S4. Lack of glutamine uptake results in growth deficiency of *S. aureus* grown in GDM+Gln.

Table S1. Composition of the glucose defined media (GDM) used in this study

Ingredients	GDM	GDM+Gln	GDM+Glu	GDM+NH_3	GDM+Gln+NH_3	GDM+Glu+NH_3
**Salts (g/L)**						
KCl	15	15	15	15	15	15
NaCl	47.5	47.5	47.5	47.5	47.5	47.5
$MgSO_4$ 7H2O	6.5	6.5	6.5	6.5	6.5	6.5
$(NH4)_2SO4$	0	0	0	20	20	20
Tris	60.5	60.5	60.5	60.5	60.5	60.5
**Carbon source (g/L)**						
Glucose	25	25	25	25	25	25
**Amino acids (mg/L)**						
L-Arg	50	50	50	50	50	50
L-Pro	10	10	10	10	10	10
L-Gln	0	100	0	0	100	0
L-Glu	0	0	100	0	0	100
L-Val	80	80	80	80	80	80
L-Thr	30	30	30	30	30	30
L-Phe	40	40	40	40	40	40
L-Leu	90	90	90	90	90	90
L-Gly	50	50	50	50	50	50
L-Ser	30	30	30	30	30	30
L-Asp	90	90	90	90	90	90
L-Lys	50	50	50	50	50	50
L-Ala	60	60	60	60	60	60
L-Trp	10	10	10	10	10	10
L-Met	10	10	10	10	10	10
L-His	20	20	20	20	20	20
L-Ile	30	30	30	30	30	30
L-Tyr	50	50	50	50	50	50
L-Cystine	20	20	20	20	20	20
**Vitamins (mg/L)**						
Biotin	0.1	0.1	0.1	0.1	0.1	0.1
Thiamine	2	2	2	2	2	2
Nicotinic acid	2	2	2	2	2	2
Calcium pantothenate	2	2	2	2	2	2
**Metals (mg/L)**						
$CaCl_2$ $2H_2O$	22	22	22	22	22	22
KH_2PO_4	140	140	140	140	140	140
$FeSO_4$ $7H_2O$	6	6	6	6	6	6
$MnSO_4$ H_2O	7.58	7.58	7.58	7.58	7.58	7.58
Citric acid	6	6	6	6	6	6

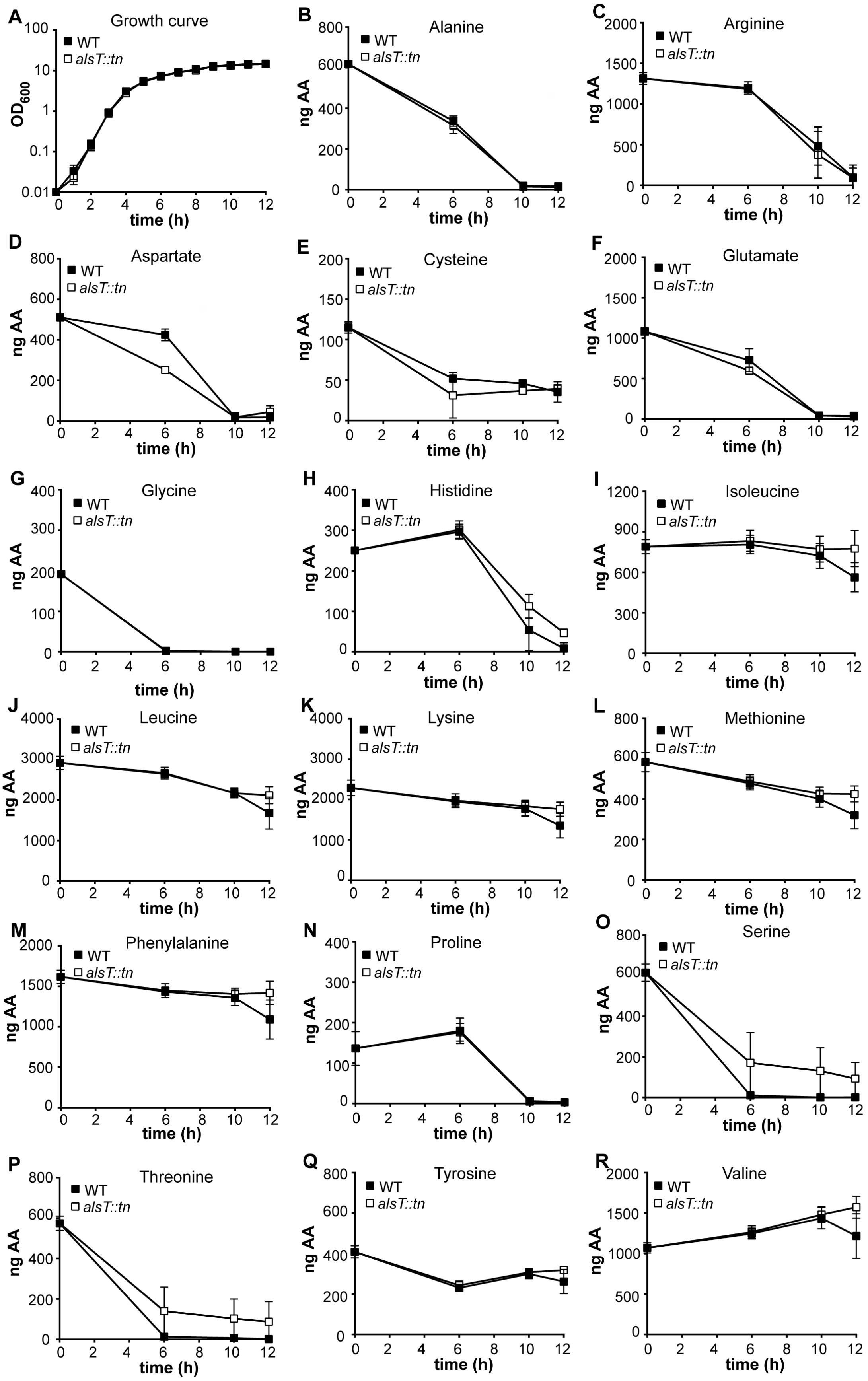
A
Growth curve
WT
alsT::tn
OD600
time (h)
B
Alanine
WT
alsT::tn
ng AA
time (h)
C
Arginine
WT
alsT::tn
ng AA
time (h)
D
Aspartate
WT
alsT::tn
ng AA
time (h)
E
Cysteine
WT
alsT::tn
ng AA
time (h)
F
Glutamate
WT
alsT::tn
ng AA
time (h)
G
Glycine
WT
alsT::tn
ng AA
time (h)
H
Histidine
WT
alsT::tn
ng AA
time (h)
I
Isoleucine
WT
alsT::tn
ng AA
time (h)
J
Leucine
WT
alsT::tn
ng AA
time (h)
K
Lysine
WT
alsT::tn
ng AA
time (h)
L
Methionine
WT
alsT::tn
ng AA
time (h)
M
Phenylalanine
WT
alsT::tn
ng AA
time (h)
N
Proline
WT
alsT::tn
ng AA
time (h)
O
Serine
WT
alsT::tn
ng AA
time (h)
P
Threonine
WT
alsT::tn
ng AA
time (h)
Q
Tyrosine
WT
alsT::tn
ng AA
time (h)
R
Valine
WT
alsT::tn
ng AA
time (h)

Fig. S1: Growth and amino acid consumption analysis to determine the function of AlsT. (A) Bacterial growth curves. *S. aureus* strains LAC* (WT) and LAC**alsT::tn* (*alsT::tn*) were grown in TSB medium and OD_{600} readings determined at hourly intervals and the average values and SDs from three biological replicates plotted. (B-R) Quantification of amino acid levels in culture supernatants. Spent medium samples from the cultures shown in panel A were prepared at the 0, 6, 10 and 12 h time points and the amino acid level determined as previously described using an amino acid analyzer (Halsey *et al.*, 2017). Amino acids analyzed are indicated above each panel. The average values and SDs from three biological replicates were plotted.

```
SAUSA300_1252    1 MKDFDSLIPGWFKEFVHVGTDLIWSQYLIGLLLTAGFFFTISSKFVQLRMLPEMFRALVERPETLEDGKK
SAUSA300_0914    1 -----------MIEKLVTFLNEVVWSKPLVYGLLITGVLFTLRMRFFQVRHFKEMIRLMFQGEK----SPN
AlsT             1 -------MESFFNSLINIPSDFIWK-YLFYILIGLGLFFTIRFGFIQFRYFIEMFRIVGEKPE----GNK
GlnT             1 -------MQQILEHIVGIANDLLWSKLLIVLLLSFGIYFTFRLKFLQVRMLKEMVRVLREGAA--SRSKN
YrbD             1 ------------MADFVASLNAVLWSTPVIYILLGIGFAFSIMTRFLQVRHLKEMIVQMFKGKS----SEA
YflA             1 -------MER-LLVWIEHISDWLWGPPLIILLTGTGLYFTILLKGFQFRYPLYIFKQTIGSVGKKPKGEG
consensus        1                .    . .*   .. .*.  *  *..  .. * *   .. . .

SAUSA300_1252   71 GISPFQAFAISAGSRVGTGNIAGVATAIVLGGPGAVFWMWVIAFIGAASAFIEATLAQVYKVHDKDGGFR
SAUSA300_0914   57 GISSFQAIAMSLAGRVGTGNIVGVSTAIFIGGPGAVFWMWITAFLGASSAFIESTLGQIFKRVEN-NEYR
AlsT            59 GVSSMQAFFISAASRVGTGNLTGVALAIATGGPGAVFWMWVVAAVGMASSFVESTLAQLYKVRDG-EDFR
GlnT            62 SISPFQAFCISMAARVGTGNITGIAIAIALGGPGAIFWMWIIAIIGSASSFVESTLAQIYKVKDV-NGFR
YrbD            56 GVSSFQALSIALSGRVGTGNIAGVATAIAFGGPGAVFWMWAIAFIGAASAFVESTLAQIYKVKQD-GQYR
YflA            63 TVTPLQALTSALSSTIGAANIVGVPAAIMFGGPGAVFWMWLIALFAMAIKFSESVLAVHYREKNEQGEYV
consensus       71  ..  **  .    ..*..*. *.  **  *****.****..* .. .. *.*..*..... .     ..

SAUSA300_1252  141 GGPAYYITKGLNQKW---LGIVFAILITITFAFVFNTVQSNTIAESLNTQYNISPVITGIILAIVTAIII
SAUSA300_0914  126 GGPAYYIEYGIGGKFGKIYGIIFAIVTIISVGLLLPGVQSNAIASSMHNAIHVPQWLMGGIVVVILGLII
AlsT           128 GGPAYYIQKGLGARW---LGIVFAILITVSFGLIFNAVQTNTIAGALDGAFHVNKIVVAIVLAVLTAFII
GlnT           131 GGPAYYMEKGLNKRW---MGALFAVLITLSFGIVFNSVQSNTVSLAFENAFGTNRLTLGLILIAVFGTII
YrbD           125 GGPAYYIEKGLGIKW---FAVLFAAAALIAMAFLMPGVQSNSIAAGIQNAFGISPFVTGCGLVLLLGFII
YflA           133 GGPMYYITKGLRMKW---LGVFFSVALIVEL-IPSIMVQGNSVSVSLAETFSFNKIYAGIGIAFLIGLVV
consensus      141 ***.**. .*. . .    ...*..   .  . .   **.*..   .   .       .  .  . . ..

SAUSA300_1252  208 FGGVRSIATLSSLIVPIMAIIYIGMVLVILLFNLDQIVPMIGTIIKSAFGIEQVTGGAVGAA----VLQG
SAUSA300_0914  196 FGGVRSIANVATAVVPFMAIIYILMAVIIICINIQEVPALFALIFKSAFGLQSAFGGIVGAM----IEIG
AlsT           195 FGGLKRVVAVSQLIVPVMAGIYILIALFVVITNITAFPGVIATIVKNALGFEQVVGGGIGGI----IVIG
GlnT           198 FGGVKRIAKLAESIVVVLAVLYIGVAFFVIFSNITQLPGVLALIVKNAFGFDQAAGGALGAA----LMQG
YrbD           192 FGGVKRIANAAQMIVPFMAIGYILLSLIIIVMNVSELPAVISLIFKSAFALDSAFGGLIGMA----ISWG
YflA           199 IGGVKRIGKVTEFVVPLMAGAYAGAGLLIVLMNLSSVPAFFSLVFSNAFTSSSAVGGFAGAALAETVRWG
consensus      211 .**.. .. .   .*. .*  *. . . ..  *.  ....   . . *..     ** .*.     .  *

SAUSA300_1252  274 IKRGLFSNEAGMGSAPNAAATAAVPHPVKQGLIQSLGVFFDTMLVCTATAIMILLYSGLKFGDNAP----
SAUSA300_0914  262 VKRGLYSNEAGQGTGPHAAAAAEVSHPSKQGLVQAFSVYIDTLFVCTATALIILISGTYNVTDGTVNANG
AlsT           261 AQRGLFSNEAGMGSAPNAAATAHVSHPAKQGFIQTLGVFFDTFIICTSTAFIILLYSVTPKG--------
GlnT           264 VRRGIFSNEAGMGSAPNAAATATTSHPVKQGLIQAFGVLTDTLVICTSTAFIILFSDAY--HTPGL----
YrbD           258 VKRGIYSNEAGQGTGPHPAAAAEVSHPVKQGLVQAFSVYIDTLFVCSATAFMILFTGMYNTQAAD-----
YflA           269 FARGLYSNEAGMGTAPIAHAAAMTDHPVRQGFWSVIGIVIDTLIICTTTAFIVLASGVWTGKNASN----
consensus      281   **..***** *..* ..* *   ** .** ..   .  **. .*. ** ..*

SAUSA300_1252  340 ---------------------QGVAVTQ-----------SALNEHLGSAGGIFLTIAVTLFAFSSVVGNY
SAUSA300_0914  332 TPHLIKDGGIYVENATGKDYSGTAMYAQAGIDKAFHGSGYQFDPTFSGVGSYFIAFALFFFAFTTILSYY
AlsT           323 ---------------------DGIQVTQ-----------AALNHHIGGWAPTFIAVAMFLFAFSSVVGNY
GlnT           328 ---------------------SGIALTQ-----------ASLSSHVGSWASGFLAILILLFGFCALIGNY
YrbD           323 -------GSFIVHQLKGVE--AGPGFTQ-----------AAIDSVLPGFGAGFVAIALFFFAFTTIMAYY
YflA           335 ---------------------DPAALTT-----------AAFQHYFGSGGGYFVSVSLVFFVVSTIMVVI
consensus      351                           ..                     .  *. . .  *......  .

SAUSA300_1252  378 YYGQSNIEFLSTNRV------ILFIFRCLVVVLVFVGAVVKTETVWNTADLFMGLMAIVNIISIIGLSNV
SAUSA300_0914  402 YITETNVAYLTRNQNNQVSSIFINIARVIILFATFYGAVKTADVAWAFGDLGVGLMAWLNIIAIWILHKP
AlsT           361 YYGETNIEFIKTSKT------WLNIYRIAVIAMVVYGSLSGFQIVWDMADLFMGIMALINLIVIALLSNV
GlnT           366 YYGETNIGFLNKSKK------LIFVYRIGVLAMIVFGCVAKVQLVWDLADLFMGLMVIVNLIAIFLLSKV
YrbD           373 YIAETNIAYLARGRESKWAML---GLKLIILAATFYGTVKTASLAWALGDAGLGIMVWLNVIAIVLLAKP
YflA           373 FYGVKQAEFLFGRLAG-------HVIKFVYLAAIIIGAAGGAKAIWGVLDLALVFIVVPNVIALLLLSRK
consensus      421 . . ... ..              . .  ..     * .      *  .*. ....  .*.* . .*

SAUSA300_1252  442 AFALMKDYQKQKK--------EGKNPVFKPENLEINLFGISAWGANKYKNSDK-----
SAUSA300_0914  472 AVNALKDYEIQKKRL-----GNGYNAVYQPDPNK--LPNAVFWLKTYPERLKQARAKK
AlsT           425 AYKVYKDYAKQRK--------QGLDPVFKAKNIPG-LKNAETWEDEKQEA--------
GlnT           430 VFTALKDYTRQKK--------AGKDPVFYKDVLK-NHNGIECWPVSDTKTDTHNKQIS
YrbD           440 ALLALKDYERQKK--------QGLDPIFDPKALG--IKNADFWEKEYTHESERVS---
YflA           436 VKALYTEFFTSEQYYLKDIRKTKQKPVYPTKEAKNS----------------------
consensus      491      ...  ...         .  ...              .
```

Fig. S2. CLUSTAL Omega alignment of *B. subtilis* and *S. aureus* AlsT/GlnT homologs.

The *S. aureus* AlsT (SAUSA300_1252) protein was used as query sequence in a BLASTP search to identify homologs encoded in *S. aureus* FPR3757 and *B. subtilis* 168. Besides, AlsT (SAUSA300_1252), one additional homolog, SAUSA300_0914, was identified in *S. aureus* and four close homologs $AlsT_{BS}$, $GlnT_{BS}$, $YrbD_{BS}$ and $YflA_{BS}$ were found in *B. subtilis.* A Clustal-Omega alignment was performed with the six proteins and identical residues were shaded in dark blue and similar residues in light blue.

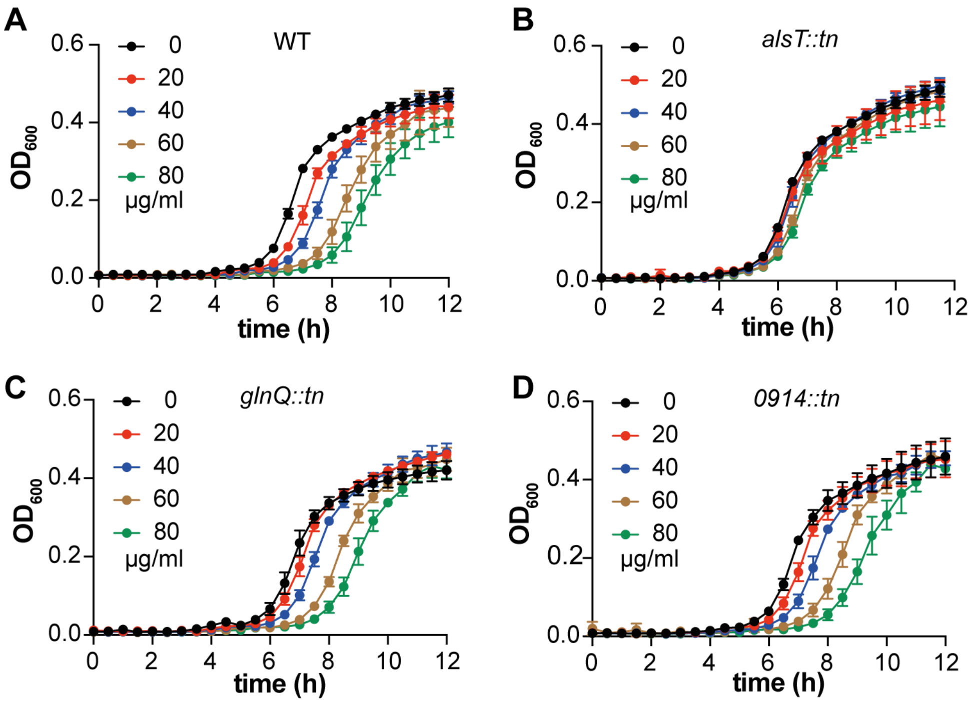

Fig. S3. LAC*_alsT::tn_ shows increased resistance to the toxic glutamine analogue γ-L-glutamyl hydrazide. (A-D). Bacterial growth curves. *S. aureus* strains (A) LAC* (WT), (B) LAC**alsT::tn* (*alsT::tn*), (C) LAC**glnQ::tn* (*glnQ::tn*) and (D) LAC**0914::tn* (*0914::tn*) were grown for 12 h in GDM+NH_3 in the presence of 0 (black), 20 (red), 40 (blue), 60 (brown) and 80 (green) µg/ml of γ-L-glutamyl hydrazide. Average OD_{600} values and SDs of three independent biological replicates were plotted.

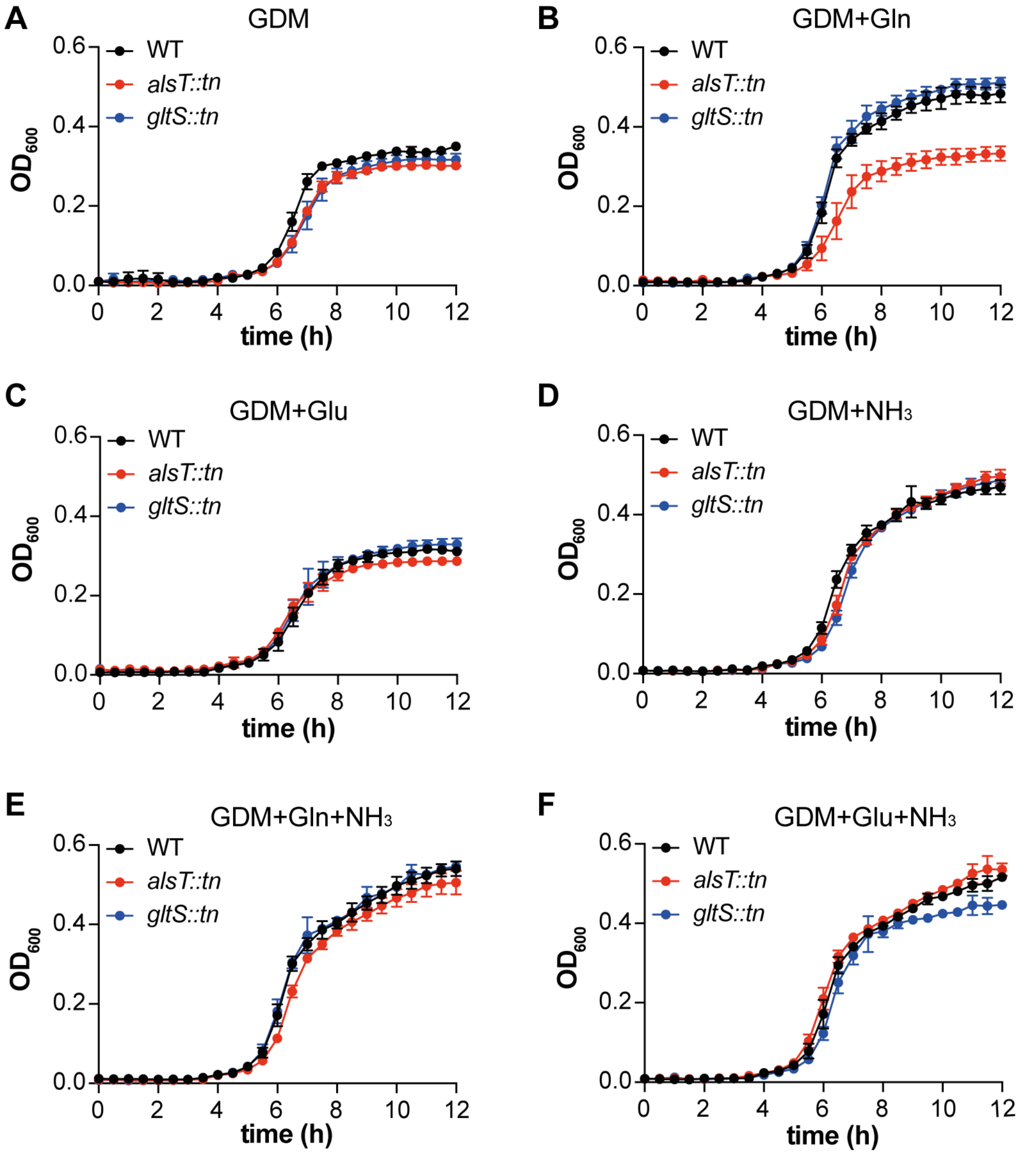

Fig. S4. Lack of glutamine uptake results in growth deficiency of *S. aureus* grown in GDM+Gln. (A-F) Bacterial growth curves. *S. aureus* strains LAC* (WT), LAC**gltS::tn* (*gltS::tn*) and LAC**alsT::tn* (*alsT::tn*) were grown in either (A) GDM, (B) GDM+Gln, (C) GDM+Glu, (D) GDM+NH_3, (E) GDM+Gln+NH_3, or (F) GDM+Glu+NH_3 for 12 h and OD_{600} readings determined. Average OD_{600} readings and SDs from three biological replicates were plotted.

REFERENCES:

Halsey, C.R., Lei, S., Wax, J.K., Lehman, M.K., Nuxoll, A.S., Steinke, L., Sadykov, M., Powers, R., and Fey, P.D. (2017) Amino Acid Catabolism in *Staphylococcus aureus* and the Function of Carbon Catabolite Repression. *MBio* **8**: e01434-01416.
